# Effectiveness of the Pilates method versus aerobic exercises in the treatment of older adults with chronic low back pain: a randomized controlled trial protocol

**DOI:** 10.1186/s12891-019-2642-9

**Published:** 2019-05-24

**Authors:** Naiane Teixeira Bastos de Oliveira, Natalia Aquaroni Ricci, Yuri Rafael dos Santos Franco, Evany Maira Espirito Santo Salvador, Isabella Cristina Barboza Almeida, Cristina Maria Nunes Cabral

**Affiliations:** 1 Masters and Doctoral Programs in Physical Therapy, Universidade Cidade de São Paulo, Rua Cesário Galeno 475, Tatuapé, São Paulo, CEP 03071-000 Brazil; 2 Department of Physical Therapy, Universidade Cidade de São Paulo, Rua Cesário Galeno 475, Tatuapé, São Paulo, 03071-000 Brazil

**Keywords:** Pilates method, Aerobic exercise, Low back pain, Elderly

## Abstract

**Background:**

Chronic low back pain is potentially disabling for older adults, and exercise is considered the best treatment. The Pilates method and aerobic exercises have been proven to be effective in pain and function improvement in patients with low back pain, but evidence in the treatment of older adults with low back pain is scarce. Therefore, the objective of this study is to investigate the effectiveness of the Pilates method compared to aerobic exercises in the treatment of older adults with chronic nonspecific low back pain.

**Methods:**

This is a randomized controlled trial with blinded assessor, to be held in a physical therapy clinic in Sao Paulo, Brazil. Seventy four patients aged 65 to 85 years with chronic nonspecific pain will be randomized into Pilates Group (*n* = 37) with exercises based on the Pilates method and Aerobic Group (n = 37) with treadmill aerobic exercise. The primary outcomes will be pain intensity and general disability, assessed eight weeks after randomization. The secondary outcomes will be: pain intensity and general disability, assessed six months after randomization; and global perceived improvement, specific disability, dynamic balance, muscle strength (gluteus maximus, gluteus medius, and lateral hip rotators), and pressure pain threshold, assessed eight weeks and six months after randomization. Therapists and patients will not be blinded.

**Discussion:**

This study has the potential to reduce pain and, consequently, improve balance and function of older adults with chronic low back pain with both therapies. However, Pilates may be more effective because the exercises are more targeted to the trunk stabilization muscles. The results of this study may provide valuable information on the effects of Pilates and aerobic exercise in older adults with chronic low back pain and contribute to a better selection of the treatment program according to the patient preference.

**Trial registration:**

ClinicalTrials.gov NCT02729779, April 6, 2016.

**Electronic supplementary material:**

The online version of this article (10.1186/s12891-019-2642-9) contains supplementary material, which is available to authorized users.

## Background

The aging process causes functional changes such as reduction in muscle mass and strength, increase in joint stiffness, and postural instability [1]. When accompanied by chronic conditions, aging is usually associated with disability [2]. Among chronic conditions, low back pain in older adults is potentially disabling [1]. Chronic low back pain is characterized by pain or discomfort lasting for more than 12 weeks between the costal margins and lower gluteal folds, with or without symptoms in the lower limbs [3]. Chronic low back pain can be classified according to the symptomatic response, and the most common is the nonspecific low back pain [3, 4]. Approximately 36% of older adults have one episode of low back pain per year [1].

Currently, physical exercise is one of the best treatment options for patients with chronic low back pain [5], as it reduces pain and disability in the short- and long-term [4] and improves balance [6]. In relation to older adults, regular exercises can minimize the deleterious physiological effects of a sedentary lifestyle, increase active life expectancy, and prevent the development and progression of chronic diseases and disabling conditions [7]. Recently published guidelines [8] for the management of chronic pain in older adults recommend strengthening, stretching, endurance and balance training to reduce functional decline, care costs, and pain intensity. Among the types of exercises that improve chronic pain, aerobic exercises (i.e., a kind of endurance training) and the Pilates method can be treatment options.

There is reliable evidence that aerobic exercises decrease pain and improve physical and psychological functioning in patients with chronic low back pain [9]. However, despite the different pathophysiologic and clinical syndromes in older adults, most of the studies about exercise in the treatment of low back pain have been conducted in younger populations [10]. Additionally, a recent systematic review found that there is a low response rate, with estimates varying from 1.4 to 45%, in interventions based on aerobic exercises in older adults compared to young patients [11]. Nevertheless, the reasons for this poor response among older adults are not well explained, which suggests that further investigation is needed.

The Pilates method is based on six basic principles: power house, concentration, control, precision, flow of movement, and breathing [12, 13]. Pilates is prescribed as a treatment for patients with chronic low back pain because involves flexibility exercises, and strength and stability exercises of the deep abdominal muscles, with more control of the movement [14, 15]. A recent systematic review in adult patients with chronic low back pain [16] showed that the Pilates method is more effective than minimal intervention (i.e. usual care or an educational booklet) in the short- and medium-term (less than three months after randomization, and at least three months and less than 12 months after randomization, respectively) for pain and disability. Similar conclusions were shown by other recent systematic reviews [17, 18]. However, Pilates is no more effective than other types of exercise (i.e. cycling or McKenzie method) for pain and disability in the short- and medium-term [16]. A systematic review about Pilates for older adults suggests that is difficult to establish its effects because there is a lack of studies with high methodological quality [19].

Exercise is indicated for chronic pain management in older adults, and studies show positive effects of aerobic exercise [9] and Pilates [16] in the improvement of symptoms of chronic low back pain in adults. Although some studies have investigated the effects of the Pilates method on the older adult population [19] with chronic low back pain [20], there is no comparison in the literature between the Pilates method and aerobic exercises in older adults with chronic nonspecific low back pain. Moreover, randomized controlled trials including older adults with chronic low back pain are scarce, limiting the generalization of the results and generating uncertainties about the efficacy of the treatments studied in this age group [8, 21]. The purpose of this study is to evaluate the effectiveness of the Pilates method compared to aerobic exercise in improving pain and disability in older adults with chronic nonspecific low back pain.

## Methods

### Study design

This will be an assessor-blinded, 2-arm, randomized controlled trial.

### Study setting

The study will be conducted at a physical therapy outpatient clinic in Sao Paulo, SP, Brazil.

### Eligibility criteria

Older adults aged 65 to 85 years, of both sexes, with chronic nonspecific low back pain and pain intensity equal to or greater than 3 points in the last seven days in the Pain Numerical Rating Scale [22] will be included. The exclusion criteria will consider current and past conditions as follows: absolute contraindication to physical activity (changes in electrocardiogram or myocardial infarction, unstable angina, uncontrolled arrhythmia, severe symptomatic aortic stenosis, uncontrolled symptomatic heart failure, acute pulmonary embolism or pulmonary infarction, acute myocarditis or pericarditis, suspected or present dissecting aneurysm, acute systemic infection accompanied by fever, body aches, or swollen lymph nodes) [23]; severe spine disease (fractures, tumors, inflammatory diseases, ankylosing spondylitis, and radicular conditions of the spine confirmed by neurological tests); previous or scheduled surgeries of the spine; severe cardiorespiratory diseases; cancer; dependent walking (with use of walking aid or assistance from another person); physical therapy treatment for low back pain in the last six months; regular physical activity, defined as physical leisure activities for 30 min three times or more per week for the last two weeks [24]; and cognitive deficit. Cognitive deficit will be screened using the Mini-Mental State Exam, with a cut-off point of less than 13 for illiterate participants, 18 for participants with elementary education, and 26 for participants with secondary and/or higher education [25]. Eligibility criteria will be screened by a trained student by telephone, with exception of cognitive deficit and nerve root compromise, which will be screened by the blinded assessor.

### Assessment

Before assessment, written consent will be obtained from the patients. A blinded assessor, who is a physical therapist with five years of clinical experience, will evaluate the eligible participants in person. The evaluation will consist of: personal data, clinical data, and evaluation of outcomes. The initial assessment will be conducted prior to the random distribution of the patients into the treatment groups, in a reserved place. The assessment of the outcomes will be repeated eight weeks and six months after randomization in the same place. To prevent loss of follow-up, patients will be asked to be available nearly the dates of follow-ups.

The primary outcomes will be pain intensity and disability, assessed eight weeks after randomization. Secondary outcomes will be as follows: global perceived improvement, specific disability, dynamic balance, muscle strength (gluteus maximus, gluteus medius, and hip lateral rotators), and pressure pain threshold, assessed eight weeks and six months after randomization. Pain intensity and disability assessed six months after randomization will be considered as secondary outcomes too. All the questionnaires/scales used in this study show adequate measurement properties [22, 26, 27].

The pain intensity perceived by the patient in the last seven days will be evaluated by the 11-point Pain Numerical Rating Scale [22], with zero being “no pain” and 10 being “pain as bad as could be”. This scale will also be applied daily before each treatment session in relation to pain intensity in the last 24 h. The pain assessment will be conducted verbally.

Disability associated with low back pain in the last 24 h will be assessed using the Roland Morris Disability Questionnaire [22, 26, 27], consisting of 24 items that describe daily activities that patients often report difficulty performing due to low back pain. The answers are yes/no, and each affirmative answer is worth one point. The final score is calculated by adding all the points. The higher the score is, the greater the limitation.

The global perceived improvement comparing the onset of symptoms to the last few days will be assessed using the Global Perceived Effect Scale [22]. It is an 11-point numerical scale (− 5 to 5), with − 5 being “vastly worse”, 0 “no change”, and 5 “completely recovered”.

Specific disability will be assessed using the Patient-Specific Functional Scale [22], which evaluates three main activities that the patient has difficulty or inability to perform due to chronic low back pain. Each activity is evaluated on an 11-point scale, with zero being “unable to perform the activity” and 10 being “able to perform the activity at pre-injury level”. The average score for the activities will be calculated, and the higher the score, the greater the specific capacity. In addition, the detailed description of each activity will be collected to enhance the presentation of the activities that are generating disability.

Dynamic balance will be assessed by the Sit-to-Stand Test and the 10-Meter Walk Test. These tests show adequate reproducibility [28] and detect meaningful changes in physical performance measures [29]. In the Sit-to-Stand Test [28, 30], the participant will sit on a chair with a backrest and upper limbs crossed over the trunk. The test instruction is: “Please stand up and sit down five times as quickly as possible”. The time taken to perform the activity (in seconds) will be measured by chronometer. The 10-Meter Walk Test [29, 31, 32] (fast and normal) requires a 10-m straight hallway. Markers will be placed at the end of the second meter and at the beginning of the eighth meter of the path to eliminate acceleration and deceleration components. The test will be performed twice, and the participant will wear his usual footwear. First, normal walking (self-selected speed) will be evaluated, followed by fast walking (maximal speed without running). The assessor will measure the walking time by starting the stopwatch when the participants’ lower limb passes the first marker (end of the second meter) and stopping the watch as soon as they cross the second marker (beginning of the eighth meter). The verbal command for normal and comfortable walking will be: “When I say go, walk at your usual comfortable pace until I tell you to stop”. The verbal command for fast walking will be: “When I say go, walk as fast as you can, safely and without running, until I tell you to stop”.

Isometric muscle strength will be assessed using a dynamometer (Lafayette Instrument Company, Indiana, United States). The gluteus medius, gluteus maximus, and lateral hip rotators will be assessed bilaterally, since these muscles are directly related to pelvic stabilization [33]. The gluteus medius muscle will be evaluated with the participant in lateral decubitus with the test leg upwards, and hip in neutral adduction and extension [34]. Resistance to movement will be applied using a non-elastic strap and the handheld dynamometer, which will be 5 cm above the lateral epicondyle of the femur. The gluteus maximus muscle will be evaluated with the patient in the ventral decubitus position with hip neutral and knee flexed at 90 degrees [35]. Resistance to movement will be applied using a non-elastic strap and the handheld dynamometer, which will be 5 cm above popliteal region. The hip lateral rotator muscles will be assessed with the participant sitting on the stretcher with hips and knees flexed at 90 degrees, upper limbs crossed over the trunk, and hip positioned in slight lateral rotation with the medial malleolus aligned with the midline of the body [35]. Resistance to movement, using a non-elastic strap and the handheld dynamometer, will be applied 5 cm above the medial malleolus. Two submaximal force tests will be performed to familiarize the participant with each test position, followed by two repetitions with maximum isometric contraction for each muscle group and the analysis will use the average of the two maximum contractions [36]. The intrarater reliability of isometric muscle strength was tested prior to data collection and was adequate (ICC above 0.81 for the three muscular tests).

Pressure pain threshold evaluates the lowest stimulus in which a patient perceives pain [37] and will be measured using a digital pressure algometer (Somedic Inc., Hörby, Sweden®). For the algometry, marks will be made with a tape measure and pen, with the participant sitting on a chair without backrest, so that two points are marked bilaterally [38]: the first mark will be 5 cm lateral to the spinous process of L3 [39] and the second will be 5 cm lateral to the spinous process of L5 [40]. A third mark will be made on the anterior tibial muscle of the right lower limb in order to have a control [41, 42]. To measure pressure pain threshold, the circular algometer probe, which is 1 cm^2^ in area, will be positioned at a 90-degree angle on the skin and will be pressed with approximately 50 kPa/s of speed. The instruction given to the participants will be to push the button when the feeling of pressure or discomfort becomes painful. The measurements in kPa will be repeated three times at each point with a 30-s interval between measurements [43]. The average of the three measurements will be used for data analysis. If the participant does not report pain at a pressure of 1000 kPa, the test will be interrupted, and this value will be adopted as the pressure pain threshold. The intrarater reliability of the pressure pain threshold was tested prior to data collection and was adequate (ICC = 0.92).

### Randomization and interventions

Randomization will be performed by a researcher not involved in the recruitment, assessment, or treatment of the participants by means of a random numerical sequence generated by Microsoft Excel 2010, before the beginning of the study. Treatment allocation will be concealed through sealed, opaque envelopes sequentially numbered. After the baseline assessment, one of the therapists responsible for the intervention will open the sealed envelope to identify the group to which the participant was allocated (Pilates Group or Aerobic Group). This is a single-blind study because only the assessor will be blind to the allocation of the participants in the treatment groups. Due to the nature of the interventions, it will not be possible to blind participants and therapists. At the end of the study, the assessor will say to which group he believes the participants were allocated in order to test the blinding of the study.

Both groups will receive 16 individual, twice-weekly exercise sessions lasting 60 min each over a period of 8 weeks. To improve adherence, participants will receive a calendar with the sessions date and the available days for missed sessions. This will ensure that the intervention period and the make-up sessions do not exceed 10 weeks, when the treatment will end, independently of the number of sessions achieved. Additionally, in São Paulo, older adults aged above 60 years do not pay for public transportation, which could ensure participants to come to the intervention site. Sessions will be supervised by two physical therapists. The Pilates Group will be supervised by a physical therapist certified in Pilates with four years of experience with the method, and the Aerobic Group will be supervised by a physical therapist specialized in Sports Physical Therapy with one year and six months of experience and specifically trained with the proposed exercises. Patient chart will be audited monthly for protocol deviations by an independent researcher. The treatments are described in Table 1. Patients will be asked to not look for other treatments outside the trial during the intervention period. Adverse events will be collected during the intervention period. Table 2 shows the timeline of the study.

**Table 1:** Description of the interventions

Group	Description
Pilates	The Pilates Group will undergo a specific exercise program including modified [38] mat-based and equipment-based Pilates exercises. In the first session, participants will receive basic training on the Pilates method and power house activation, which is the isometric contraction of the transverse abdominis, multifidus, and pelvic floor muscles during expiration [12, 44]. Power house activation will always be combined with the exercises in order to stabilize low back. The session will be divided into: warm-up and global stretching (5 min), exercises for the upper and lower limbs, abdomen, and spine (45 min), global stretching (5 min), and relaxing local massage (5 min). There will be a minimum of five exercises and a maximum of 15 exercises in each session. Each exercise will be selected individually, according to the objective of the treatment and preference of the patient and adapted if patient’s pain worsens. For example, if the objective of the treatment is to strengthen gluteus muscles and patient cannot get into a desired position or feels pain, the exercise will be adapted to another position or equipment without changing its objective. The exercises will be modified so that they can be performed at three levels of difficulty: basic, intermediate, and advanced (Additional file 1: Appendix 1 and Additional file 2: Appendix 2). In the first session, the patients will perform the basic exercises. The progress to a new level of difficulty will occur whenever postural compensations decrease, and the patient can perform eight to 10 repetitions easily and without pain.
Aerobic	The Aerobic Group will be submitted to a global stretching program (lower limbs, upper limbs, and spine with two repetitions and maintenance of each stretch for 30 s in each segment [45]) for a total of 10 min, walking on the treadmill for 20 to 40 min (intensity will be increased according to individual ability and report of pain), and relaxation with local massage for 5 minutes [7]. Training intensity will be based on the combination of heart rate (based on the maximum heart rate percentage: 208 - (0.7 x age)) [46] and the rate of perceived exertion assessed through the Borg scale [47]. A percentage of 50 to 75% of the maximum heart rate and levels between 12 and 13 (moderate intensity) of the Borg scale will be used [48].

**Table 2:** Timeline for the schedule of enrolment, interventions, and assessments

Outcomes	Enrolment	Before randomization	Intervention period (8 weeks)	8-week follow-up after randomization	6-months follow-up after randomization
Eligibility criteria	X				
Demographic data	X				
Informed consent	X				
Primary outcomes					
Pain intensity		X		X	
General disability		X		X	
Secondary outcomes					
Pain intensity			X		X
General disability					X
Global impression of improvement		X		X	X
Specific disability		X		X	X
Dynamic balance		X		X	X
Muscular strength		X		X	X
Interventions					
Pilates method treatment			X		
Aerobic treatment			X		

### Sample size

The study was designed to detect a clinically significant 2-point difference in pain intensity assessed by the Pain Numerical Rating Scale (estimate for standard deviation = 2.5) and a 5-point difference in disability assessed by the Roland Morris Disability Questionnaire (estimate for standard deviation = 6.5) [49, 50] eight weeks after randomization, using a two-tailed t-test of difference between means and assuming α = 0.05, statistical power of 80% [51], and loss to follow-up of 15%. Estimations indicated that 34 patients per group will be required using pain intensity to calculate sample size, and 37 patients per group using disability. Therefore, the biggest number resulting from sample size calculation will be considered, which corresponds to 74 patients. To reach this number, the study will be promoted in community newspapers and on the internet.

### Statistical analysis

The data collected will be stored in locked cabinets, and only the blinded assessor will have access to this information. Data will be saved in a computer spreadsheet with password protection to ensure confidentiality. To ensure no error, the spreadsheet will be monthly monitored and audited by a researcher who is blind to the participants’ group allocation and has no conflict of interest. The mean effects of the interventions and the differences between groups for all outcomes and their respective 95% confidence intervals will be calculated using linear mixed models, which will incorporate terms for the treatment groups, time (i.e. three variables will be created for the categories baseline, 8-week and 6-month follow-ups), and interaction terms (treatment groups versus time). Treatment coefficients versus time interactions will be equivalent to the estimates of the differences between groups. The analyses will follow the principles of intention to treat and no interim analyses will be performed. No additional analysis will be performed. If a patient drops out of treatment, no additional outcome will be collected. The significance level will be set at 5%, and SPSS for Windows will be used for the statistical analysis.

## Discussion

Pilates method and aerobic exercises have been shown to improve balance and reduce pain and disability in patients with chronic low back pain [9, 16, 19]. However, studies investigating the effects of Pilates on this population generally exclude older adults or consider patients aged 18 to 80 years, which is a wide range of age, making the results not specific for older adults [21]. Moreover, older adults show a lower response rate to aerobic interventions higher than the observed in younger patients [11]. Thus, the effects of the Pilates method and aerobic exercises on older adults with chronic low back pain are poorly understood. Therefore, the results of this study will help to determine if these exercises are also effective for older adults. We believe that, with the reduction in pain, there will be an improvement in balance [52] and function with both therapies. On the other hand, Pilates may be more effective for pain and disability because exercises are more targeted to the muscles of the pelvis and trunk [12, 44]. If our hypothesis is proven, this may increase the treatment options that clinicians can offer to patients, considering their preferences.

Therefore, the results of the present study will contribute to more accurate estimates of the therapeutic effects of Pilates and aerobic exercises in older adults and will be the largest randomized controlled trial in this field. The present study will be conducted with low risk of bias to obtain an eight score on the PEDro scale. Therefore, a sample calculation has been performed to identify a significant clinical effect and a pragmatic study has been proposed, taking into account the individuality of each patient through an exercise protocol (Additional file 1: Appendix 1 and Additional file 2: Appendix 2) and providing individualized therapies tailored to each patient. We believe that the results of this study may help physical therapists to make clinical decisions based on a high-quality methodological study. The results should be published in an international scientific journal after data collection.

## Additional files

Additional file 1: Mat-based exercises. The appendix 1 contains photos and description of Pilates exercises that could be performed on mat. (PDF 3667 kb)

Additional file 2: Equipment-based exercises. The appendix 2 contains photos and description of Pilates exercises that could be performed on equipment. (PDF 2285 kb)

## Abbreviations

ICC: Intraclass Correlation Coefficient
SPSS: Statistical Package for the Social Sciences
