# Supplementary material for: Effectiveness of the Pilates method versus aerobic exercises in the treatment of older adults with chronic low back pain: a randomized controlled trial protocol
Source: BMC Musculoskelet Disord. 2019 May 24;20:250. doi: 10.1186/s12891-019-2642-9 (PMC6533704; doi:10.1186/s12891-019-2642-9)
Supplement: Supplementary file 1 — Mat-based exercises. The appendix 1 contains photos and description of Pilates exercises that could be performed on mat. (PDF 3667 kb) [file 12891_2019_2642_MOESM1_ESM.pdf]

APPENDIX 1- Equipment-based exercises

Spine Exercises		
**Basic**	**Intermediate**	**Advanced**
Bridge Variant<br>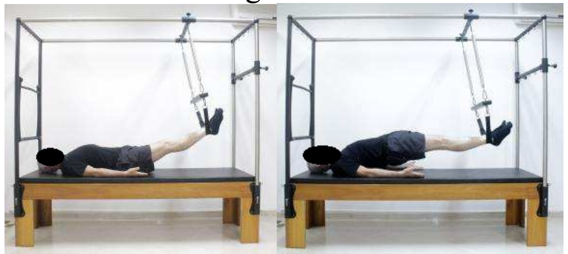<br>Objective: mobilize pelvis and spine and strengthen hamstrings and gluteus muscles.	Bridge Variant (flexed knees)<br>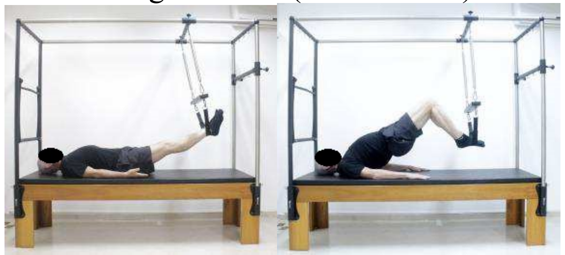<br>Objective: mobilize pelvis and spine and strengthen hamstrings and gluteus muscles.	Bridge Variant (flexed knees)<br>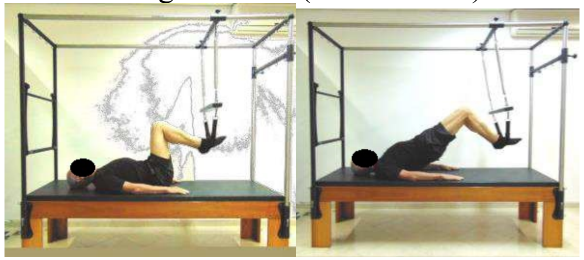<br>Objective: strengthen hamstrings, quadriceps femoris, triceps surae and gluteus muscles.
Rolling Back Down/Up (heavy spring and flexed knees)<br>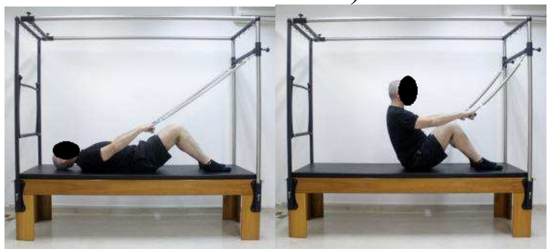<br>Objective: mobilize the spine and strengthen the rectus abdominis and external oblique muscles.	Rolling Back Down/Up (heavy spring and extended knees)<br>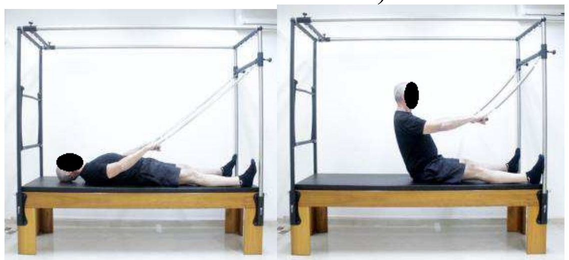<br>Objective: mobilize the spine and strengthen the rectus abdominis and external oblique muscles.	Rolling Back Down/Up (mild spring and extended knees)<br>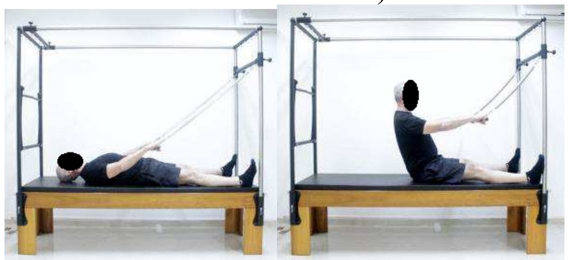<br>Objective: mobilize the spine and strengthen the rectus abdominis and external oblique muscles.
Hamstring Stretch Variant (both feet on the ground)<br>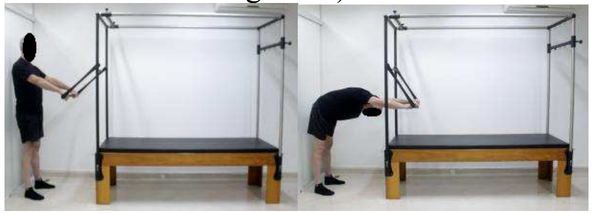<br>Objective: mobilize the spine and stretch posterior chain muscles.	Hamstring Stretch Variant (one foot in front of another)<br>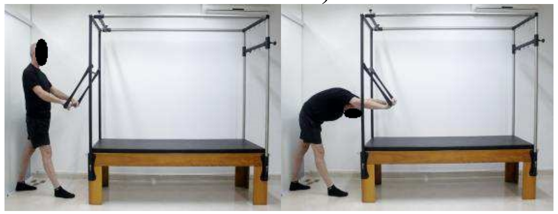<br>Objective: mobilize the spine and stretch posterior chain muscles.	Hamstring Stretch Variant (one foot on the Cadillac)<br>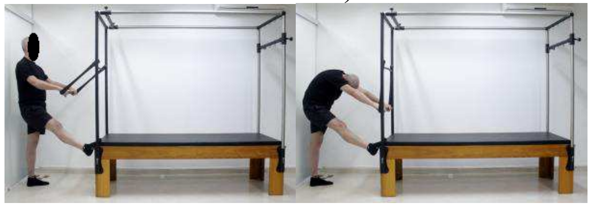<br>Objective: mobilize the spine and stretch posterior chain muscles.

Hamstring Stretch Variant (both legs on the ball)<br>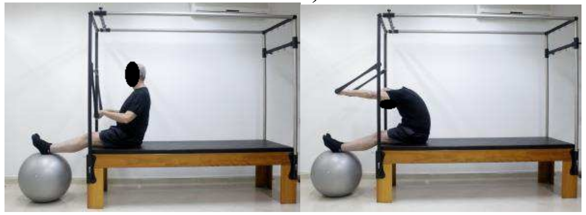<br>Objective: mobilize the spine and stretch posterior chain muscles.	Hamstring Stretch Variant (one leg on the ball and the other in the air)<br>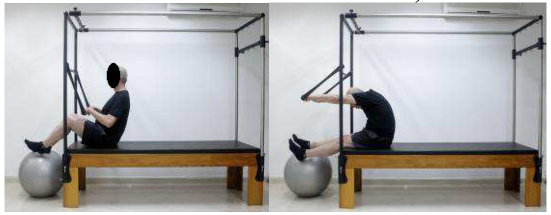<br>Objective: mobilize the spine and stretch posterior chain muscles.	Spine Stretch<br>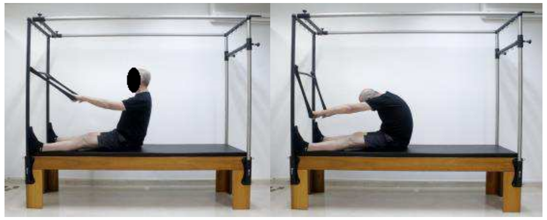<br>Objective: mobilize the spine and stretch posterior chain muscles.
Knee Stretches Round (heavy spring)<br>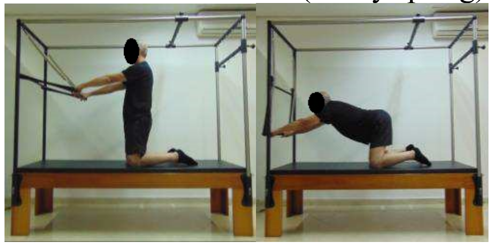<br>Objective: mobilize and provide coordination for the movements of the spine.	Knee Stretches Round (mild spring)<br>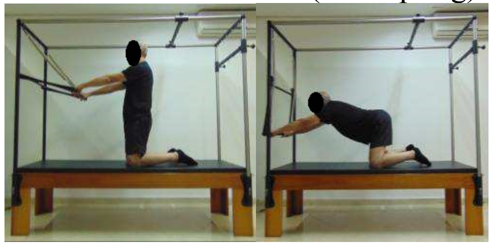<br>Objective: mobilize and provide coordination for the movements of the spine.	Knee Stretches Round (without spring)<br>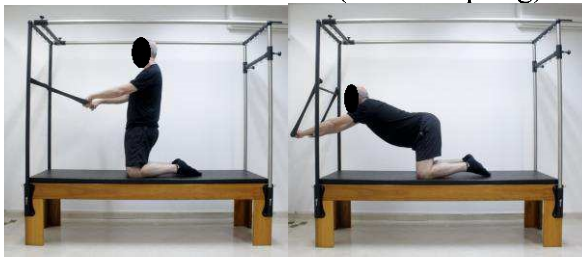<br>Objective: mobilize and provide coordination for the movements of the spine.
Swan (raising the spine)<br>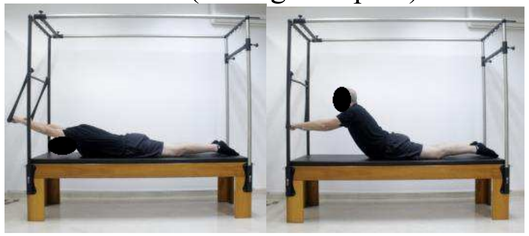<br>Objective: mobilize the spine, control the scapular elevation movement and strengthen the posterior chain muscles.	Swan (raising legs)<br>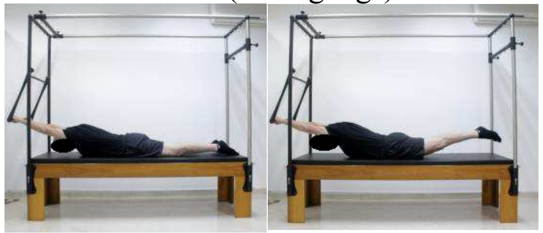<br>Objective: mobilize the spine, control the scapular elevation movement and strengthen the posterior chain muscles.	Swan (raising legs and spine)<br>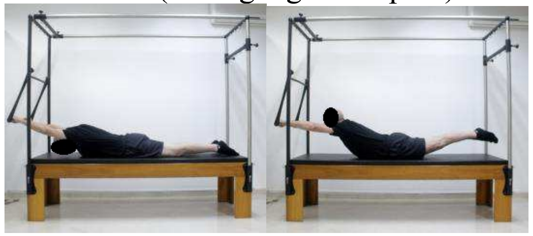<br>Objective: mobilize the spine, control the scapular elevation movement and strengthen the posterior chain muscles.

Swan Front (spine extension)<br>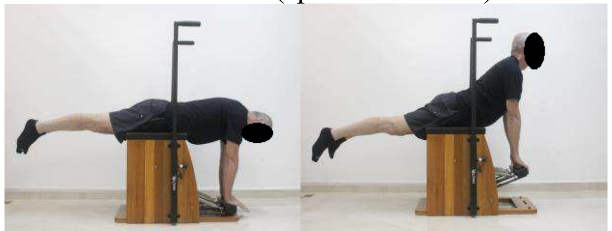<br>Objective: stretch anterior chain muscles and strengthen paravertebral muscles.	Swan Front (rotating the head)<br>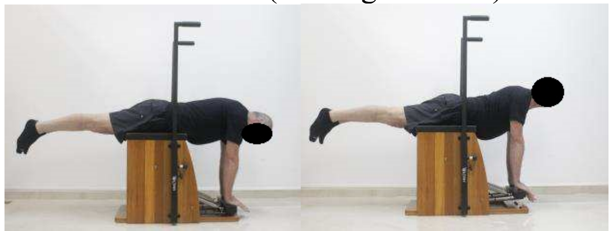<br>Objective: stretch anterior chain muscles and strengthen paravertebral muscles.	Swan Front (unilateral with spine extension)<br>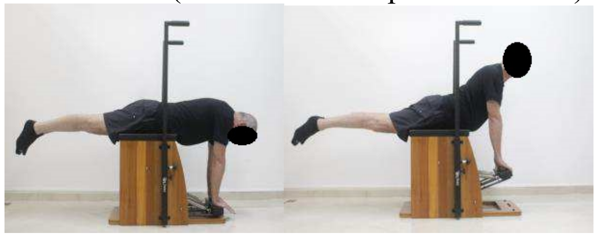<br>Objective: stretch anterior chain muscles and strengthen paravertebral muscles.
Tower (one spring)<br>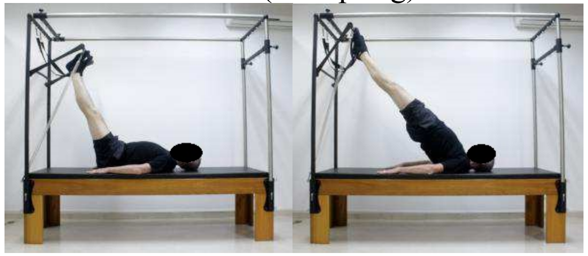<br>Objective: mobilize the spine, stretch posterior chain muscles and strengthen the abdominal, gluteus, hamstrings and triceps surae muscles.	Tower (two springs)<br>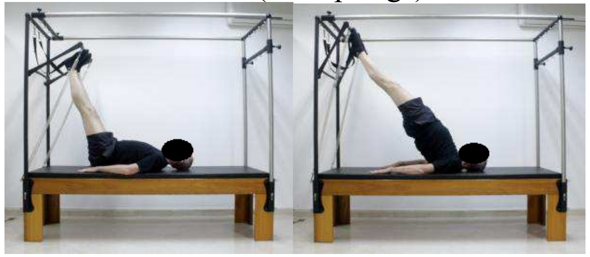<br>Objective: mobilize the spine, stretch posterior chain muscles and strengthen the abdominal, gluteus, hamstrings and triceps surae muscles	Tower (two heavy springs)<br>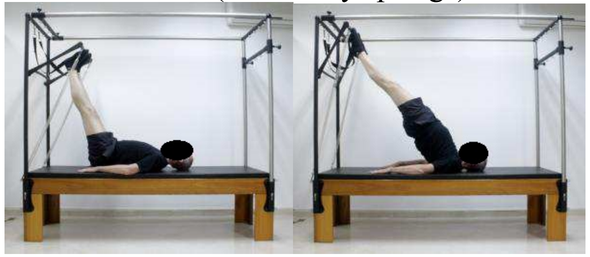<br>Objective: mobilize the spine, stretch posterior chain muscles and strengthen the abdominal, gluteus, hamstrings and triceps surae muscles.
Mermaid<br>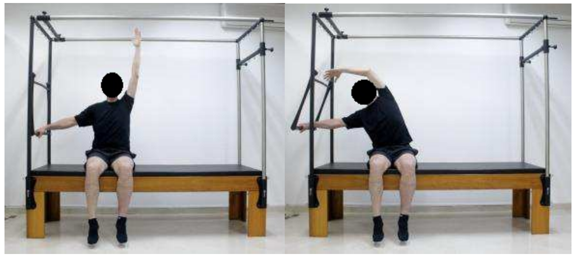<br>Objective: stretch lateral chain muscles and control scapular elevation movement.	Mermaid<br>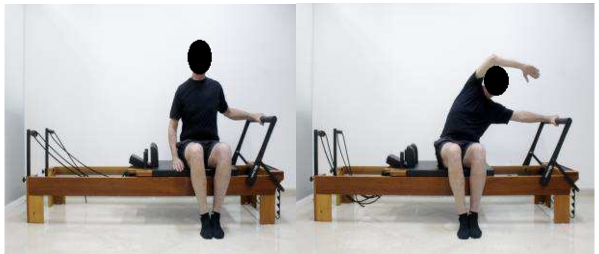<br>Objective: stretch lateral chain muscles and control scapular elevation movement.	Side Arm Sit Variant<br>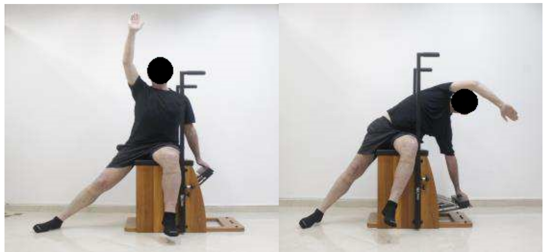<br>Objective: stretch lateral chain muscles and control scapular elevation movement.

Rolling Back Variant	Rolling Back	Swan Variant
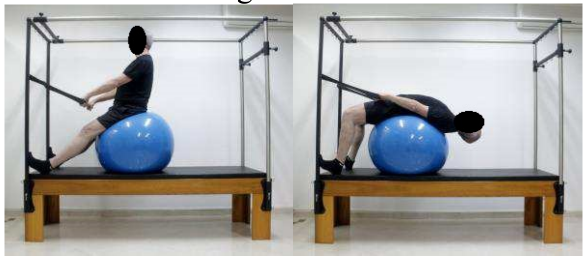	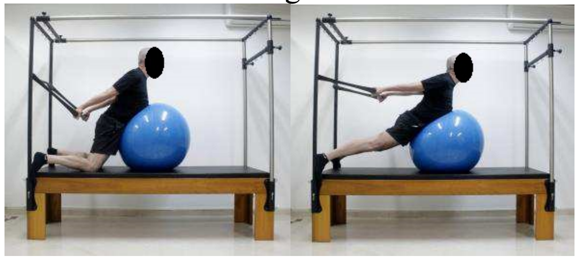	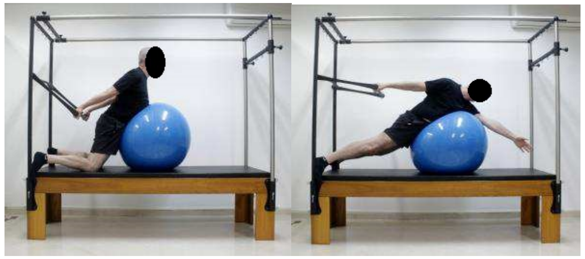
Objective: mobilize the spine and control abdominal muscles contraction associated with power house.	Objective: mobilize the spine, stretch antero-internal chain of shoulder muscles and control abdominal muscles contraction associated with power house.	Objective: mobilize the spine, control scapular elevation movement, and stretch antero-internal chain shoulder muscles.

Lower Limbs Exercises		
**Basic**	**Intermediate**	**Advanced**
Tower<br>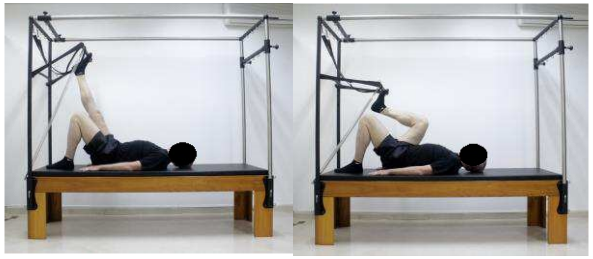<br>Objective: mobilize the spine, stretch posterior chain muscles and strengthen quadriceps femoris, hamstrings and gluteus maximus muscles.	Tower<br>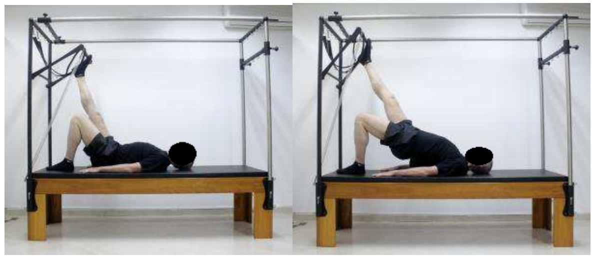<br>Objective: mobilize the spine, stretch posterior chain muscles and strengthen quadriceps femoris, hamstrings and gluteus maximus muscles.	Tower (heavy spring)<br>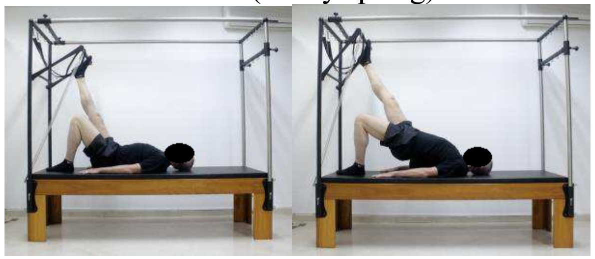<br>Objective: mobilize the spine, stretch posterior chain muscles and strengthen quadriceps femoris, hamstrings and gluteus maximus muscles.
Running<br>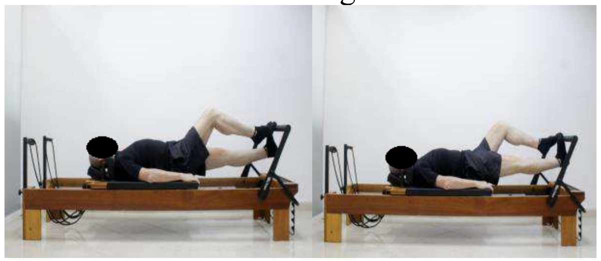<br>Objective: stretch posterior chain muscles and strengthen quadriceps femoris, gluteus maximus and triceps surae muscles.	Tower<br>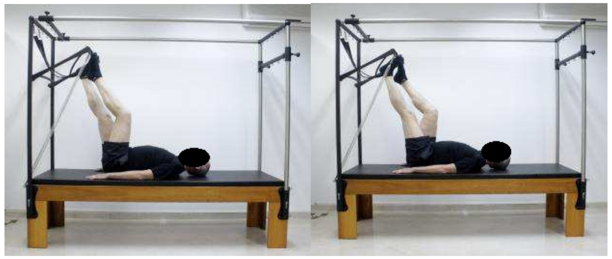<br>Objective: stretch posterior chain muscles and strengthen quadriceps femoris, gluteus maximus and triceps surae muscles.	Stomach Massage Series<br>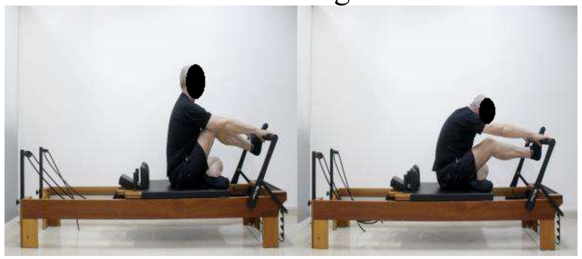<br>Objective: mobilize the spine, stretch posterior chain muscles and strengthen the quadriceps femoris and triceps surae muscle.
Footwork<br>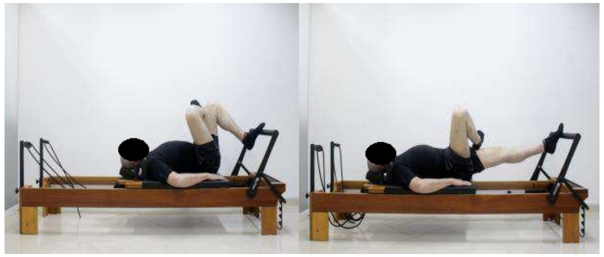<br>Objective: stretch gluteus maximus and piriformis muscles and strengthen the quadriceps femoris muscle.	Tower<br>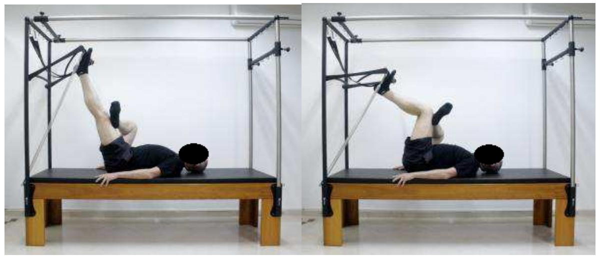<br>Objective: stretch gluteus maximus and piriformis muscles and strengthen the quadriceps femoris muscle.	Tower (heavy spring)<br>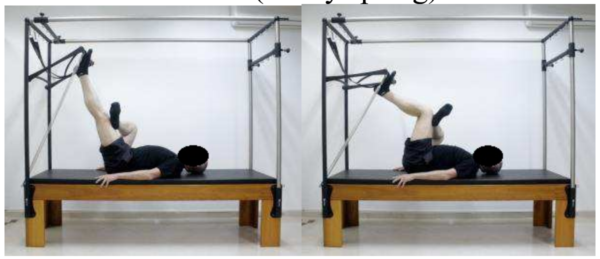<br>Objective: stretch gluteus maximus and piriformis muscles and strengthen the quadriceps femoris muscle.

Footwork<br>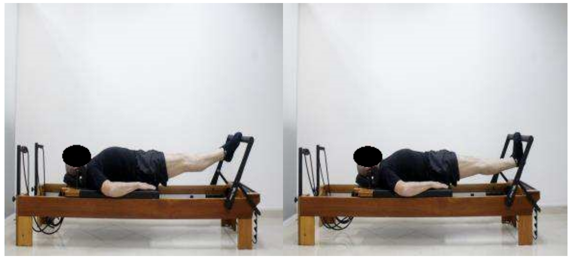<br>Objective: stretch hamstrings and gastrocnemius muscles and strengthen gastrocnemius muscles.	Achilles Stretch<br>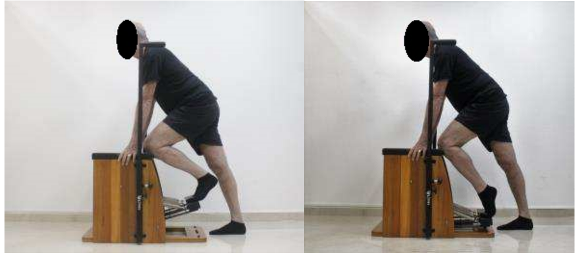<br>Objective: stretch and strengthen gastrocnemius muscles.	Tower (with both feet)<br>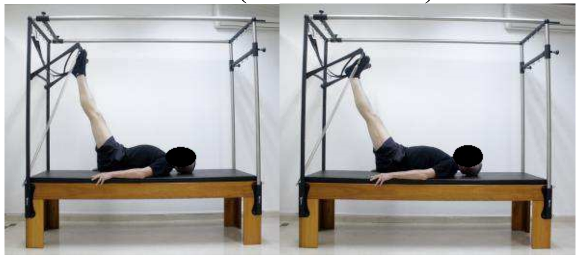<br>Objective: stretch hamstrings and gastrocnemius muscles and strengthen gastrocnemius.
Leg Series Supine Circle (mild spring)<br>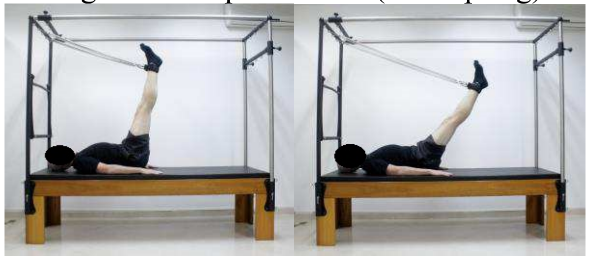<br>Objective: strengthen gluteus maximus, hamstrings, adductor magnus, adductor longus, pectin and gracilis muscles.	Leg Series Supine Circle (heavy spring)<br>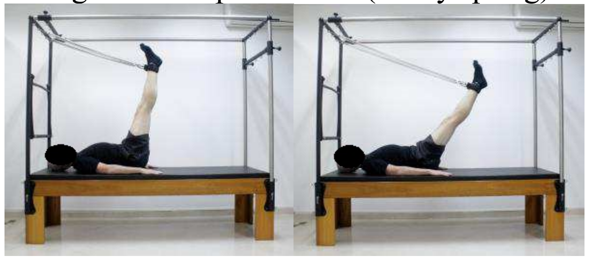<br>Objective: strengthen gluteus maximus, hamstrings, adductor magnus, adductor longus, pectin and gracilis muscles.	Leg Circles<br>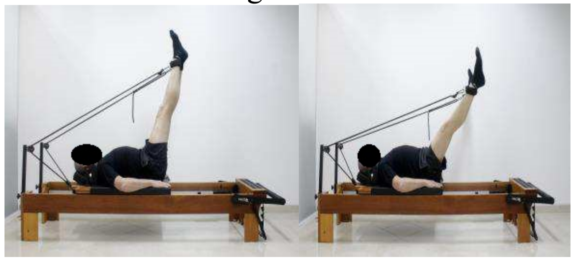<br>Objective: strengthen gluteus maximus, hamstrings, adductor magnus, adductor longus, pectin and gracilis muscles.
Leg Series Supine Lowers (bicycle)<br>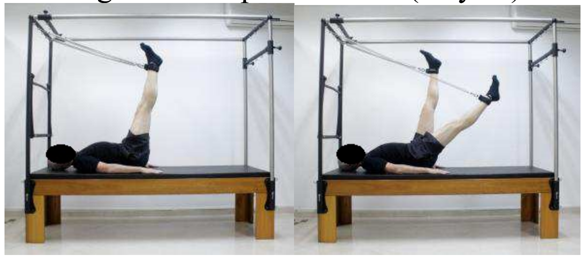<br>Objective: strengthen the gluteus maximus and hamstrings muscles.	Leg Lowers (both legs)<br>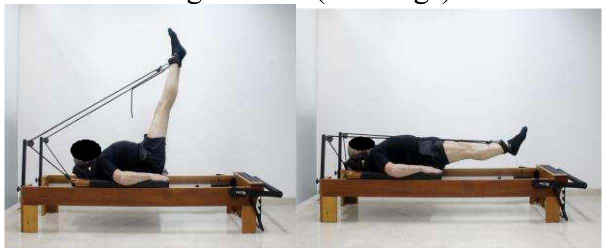<br>Objective: strengthen the gluteus maximus and hamstrings muscles.	Leg Series Supine Scissors (both legs)<br>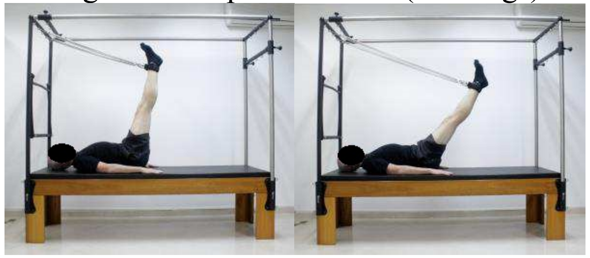<br>Objective: strengthen the gluteus maximus and hamstrings muscles.

Footwork Hells V Position<br>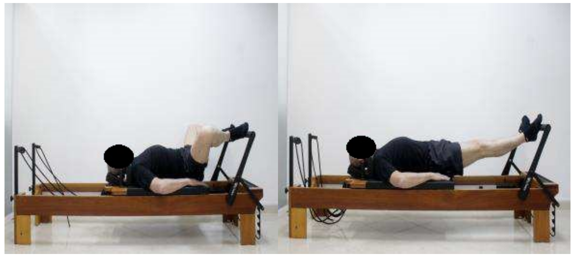<br>Objective: strengthen the quadriceps femoris, adductor magnus, adductor longus, pectin and gracilis muscles.	Leg Series Supine Frog (lower spring)<br>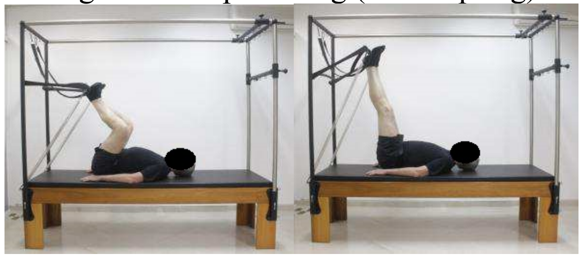<br>Objective: strengthen the quadriceps femoris, adductor magnus, adductor longus, pectin and gracilis muscles.	Leg Series Supine Frog (upper spring)<br>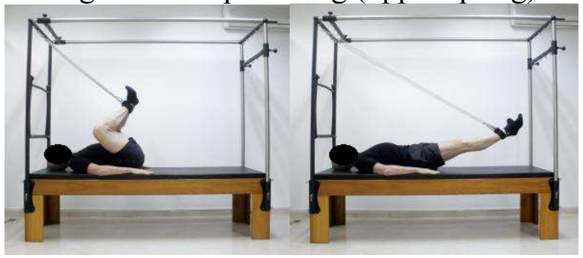<br>Objective: strengthen the quadriceps femoris, adductor magnus, adductor longus, pectin and gracilis muscles.
Footwork Double Leg Pumps<br>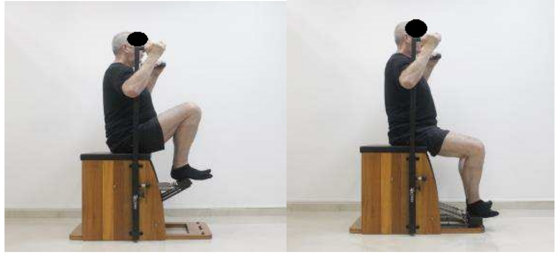<br>Objective: strengthen the quadriceps femoris and triceps surae muscles.	Pump One Leg Front<br>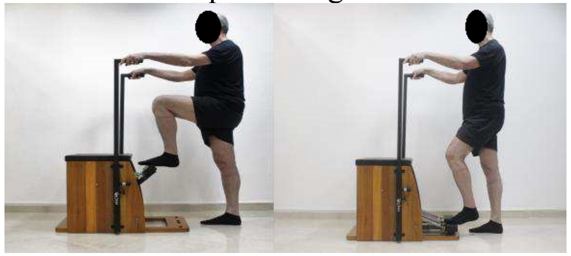<br>Objective: strengthen the quadriceps femoris and triceps surae muscles.	Pumping One Leg<br>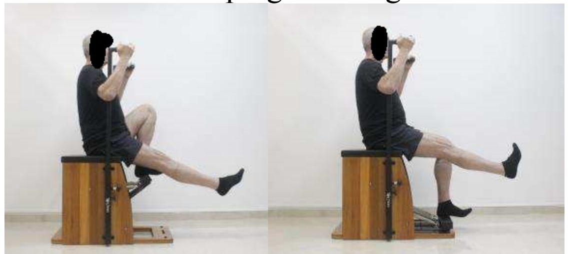<br>Objective: strengthen the quadriceps femoris, triceps surae, hamstrings and sartorius muscles.
Pump One Leg Side<br>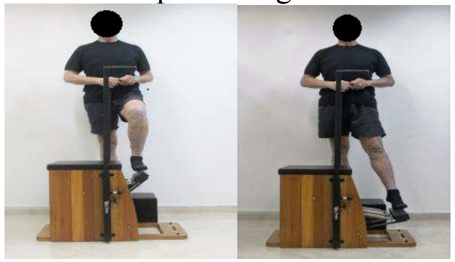<br>Objective: strengthen gluteus medius and tensor fascia lata muscles.	Side Splits (mild spring)<br>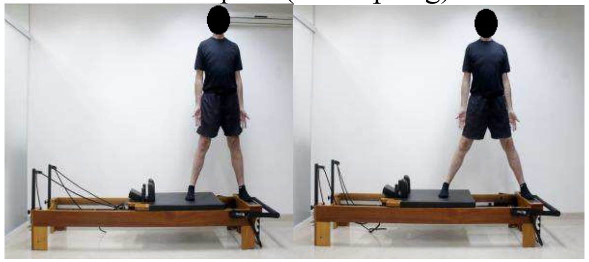<br>Objective: strengthen gluteus medius , minimum gluteus, tensor fascia lata and sartorius muscles.	Side Splits (heavy spring)<br>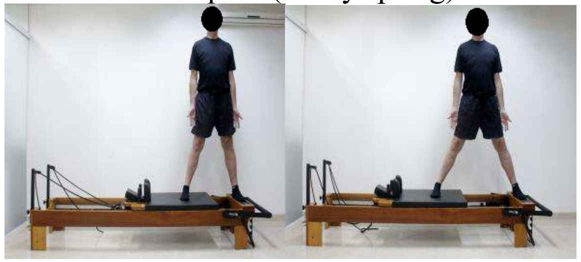<br>Objective: strengthen gluteus medius , minimum gluteus, tensor fascia lata and sartorius muscles.

Leg Series Supine (patient near the mild spring)<br>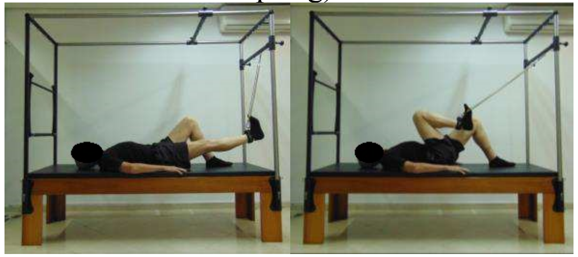<br>Objective: strengthen hamstrings, rectus femoris and rectus abdominis muscles.	Leg Series Supine (patient near the heavy spring)<br>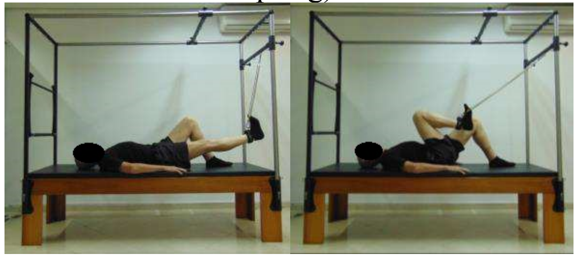<br>Objective: strengthen hamstrings, rectus femoris and rectus abdominis muscles.	Leg Series Supine (patient far from the heavy spring)<br>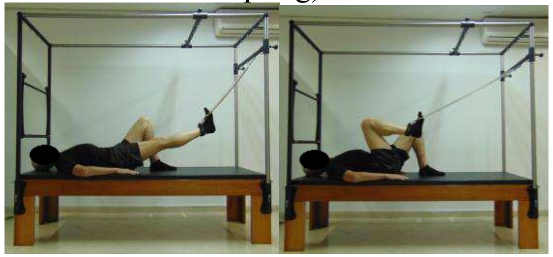<br>Objective: strengthen hamstrings, rectus femoris and rectus abdominis muscles.
Footwork Toes<br>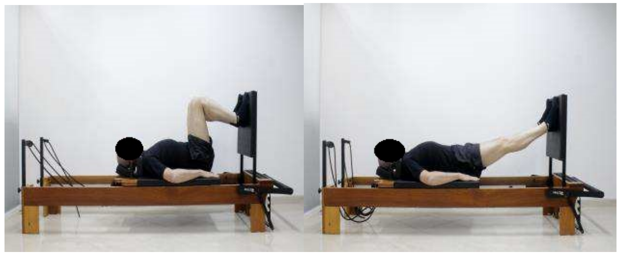<br>Objective: strengthen quadriceps femoris muscle.	Leg Series<br>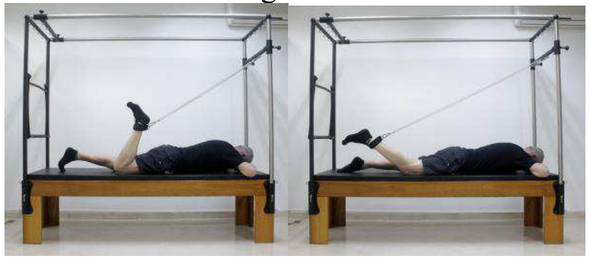<br>Objective: strengthen quadriceps femoris muscle.	Front Splints<br>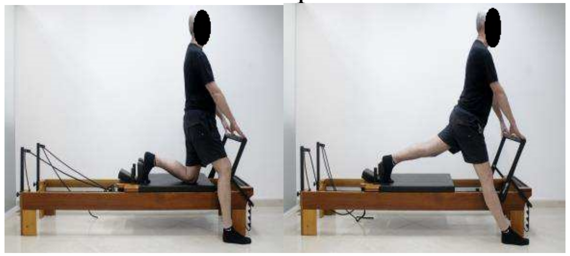<br>Objective: strengthen quadriceps femoris and gluteus muscles.
Footwork<br>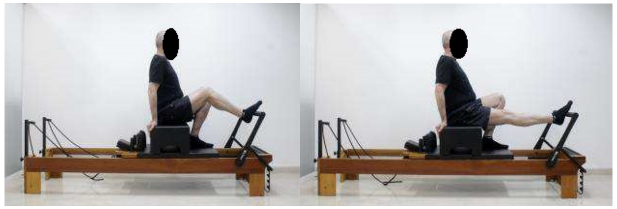<br>Objective: strengthen quadriceps femoris muscle.	Footwork<br>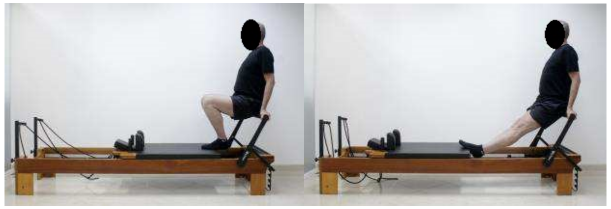<br>Objective: strengthen quadriceps femoris muscle.	Going Up-Front<br>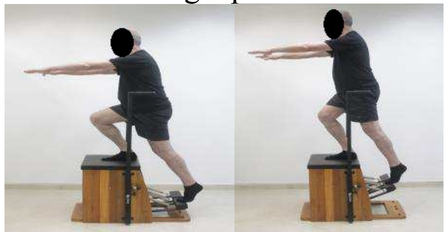<br>Objective: strengthen quadriceps femoris and gluteus muscles.

Leg Series On Side Up and Down (low spring)<br>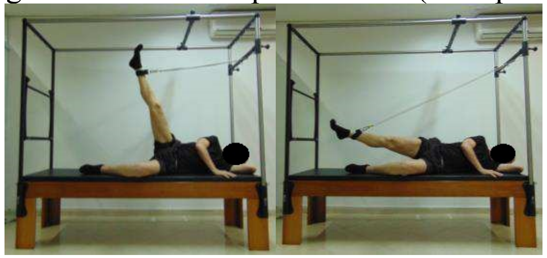<br>Objective: strengthen adductor magnus, adductor longus, adductor minimum, pectin and gracilis muscles.	Leg Series On Side Up and Down (high spring)<br>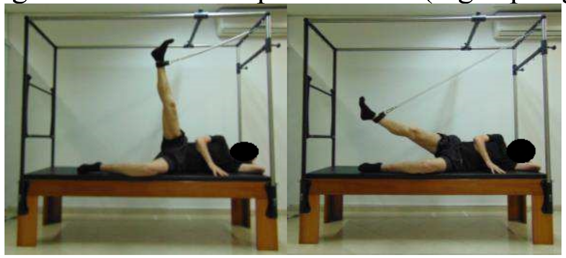<br>Objective: strengthen magnus adductor, long adductor, adductor minimum, pectin and gracilis muscles.	Scissor Leg Side Lying<br>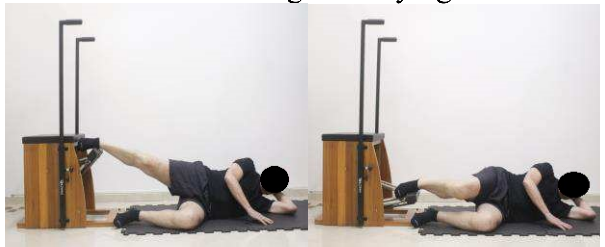<br>Objective: strengthen magnus adductor, long adductor, adductor minimum, pectin and gracilis muscles.
Stretches Front (patient and foot forward)<br>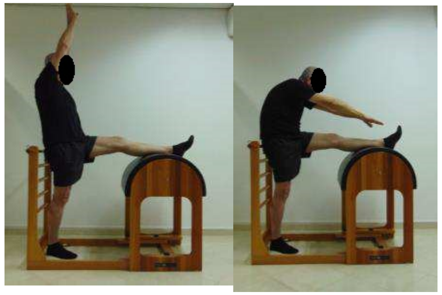<br>Objective: mobilize the spine and stretch hamstrings and triceps surae muscles.	Stretches Front (patient and foot sideways)<br>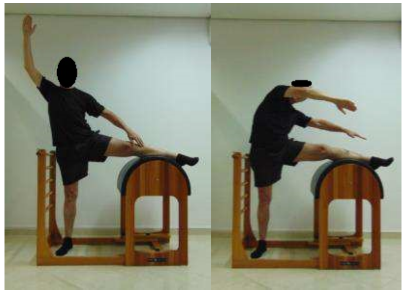<br>Objective: mobilize the spine and stretch hamstrings and triceps surae muscles.	Stretches Front (patient on the side and foot forward)<br>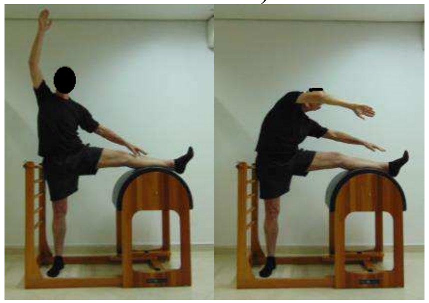<br>Objective: mobilize the spine and stretch hamstrings and triceps surae muscles.
Hamstring Stretch (sitting on Cadillac)<br>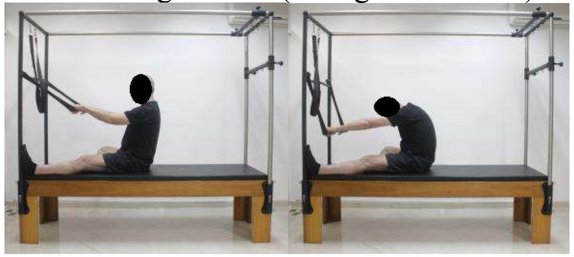<br>Objective: mobilize the spine and stretch posterior chain muscles.	Hamstring Stretch<br>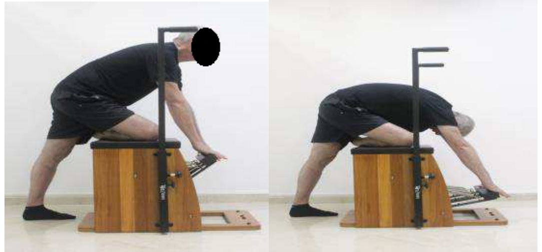<br>Objective: mobilize the spine and stretch posterior chain muscles.	Stretches Front<br>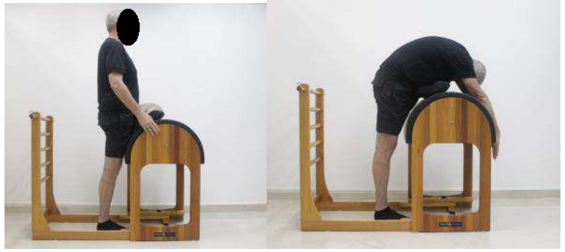<br>Objective: mobilize the spine and stretch gluteus and hamstring muscles.

Upper Limb Exercises		
**Basic**	**Intermediate**	**Advanced**
Arms Biceps (mild spring)<br>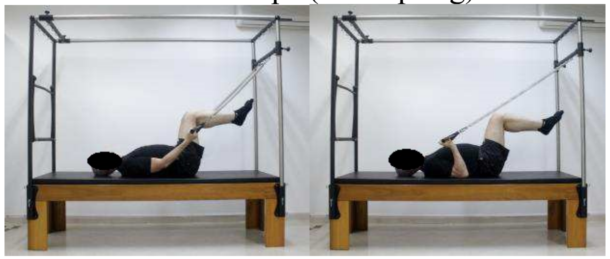<br>Objective: strengthen the brachial and brachial biceps muscles.	Arms Biceps Variant (heavy spring)<br>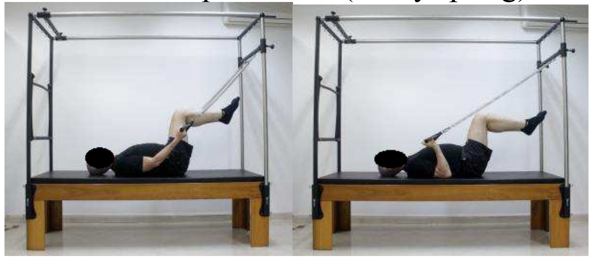<br>Objective: strengthen the brachial and brachial biceps muscles.	Arms Biceps Variant<br>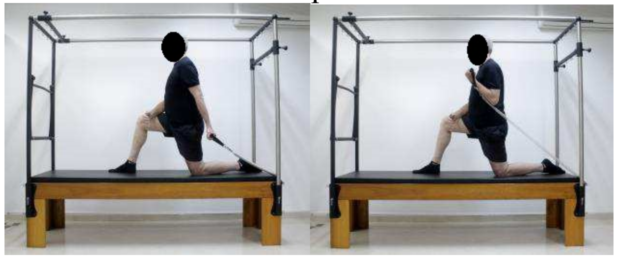<br>Objective: strengthen brachial biceps, brachial, rectus abdominis, iliopsoas and rectus femoris muscles.
Arms Biceps<br>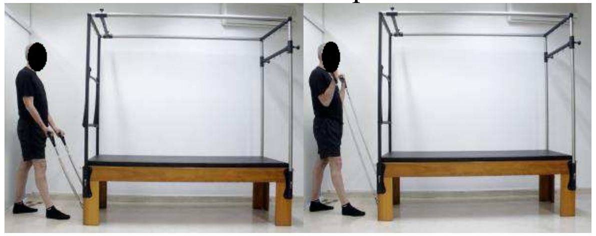<br>Objective: strengthen the brachial biceps, brachial and brachioradial muscles.	Arms Biceps<br>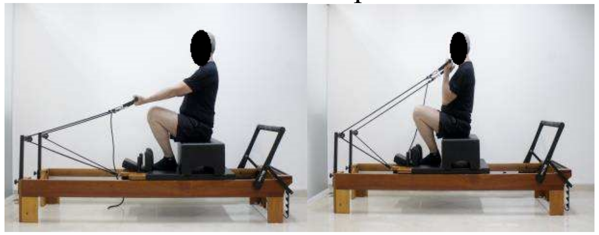<br>Objective: strengthen the brachial and brachial biceps muscles.	Arms Biceps Variant<br>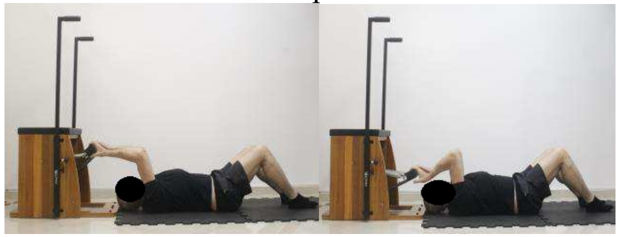<br>Objective: strengthen the brachial and brachial biceps muscles.
Arms Pulling<br>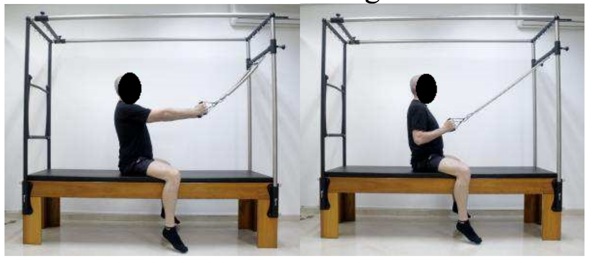<br>Objective: strengthen the rhomboid, latissimus dorsi, posterior deltoid fibers, brachial biceps, medium and inferior fibers of trapezius and teres minor muscles.	Arms Pulling<br>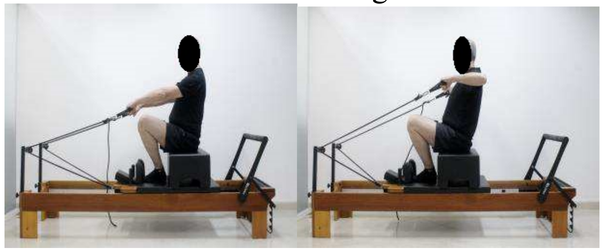<br>Objective: strengthen the rhomboid, posterior deltoid, teres minor, infraspinatus, supraspinatus and medial and inferior trapezius fibers muscles.	Arms Pulling Variant (lying)<br>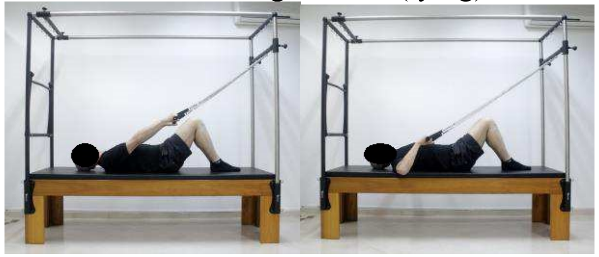<br>Objective: strengthen the rhomboid, posterior deltoid, teres minor, infraspinatus, supraspinatus and medial and inferior trapezius fibers muscles.

Arms Pull Up and Down Variant<br>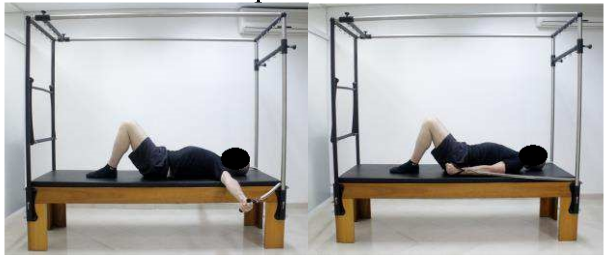<br>Objective: strengthen the pectoralis and latissimus dorsi muscles.	Arms Pull Up and Down Variant<br>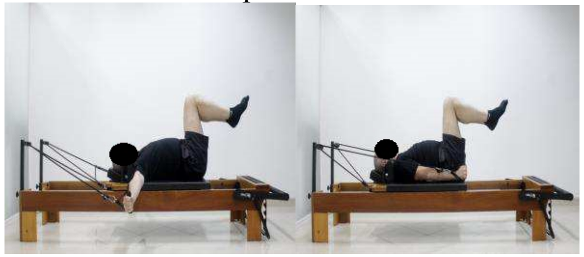<br>Objective: strengthen deltoid, latissimus dorsi, teres major, major pectoralis and coracobrachialis muscles.	Long Box Pulling Straps<br>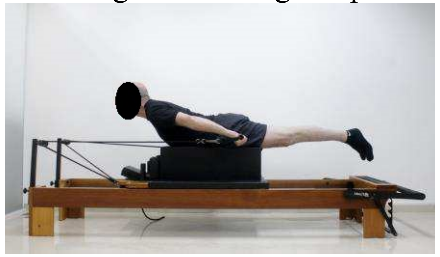<br>Objective: strengthen latissimus dorsi, teres major, pectoralis major and coracobrachialis muscles.
Arms Triceps<br>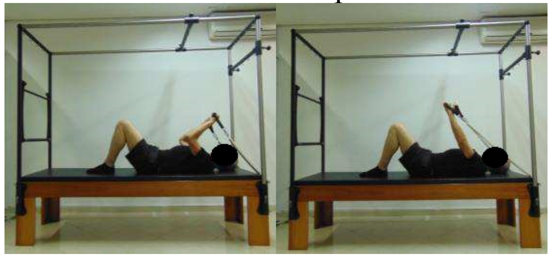<br>Objective: strengthen the triceps brachii muscle.	Arm Triceps (mild spring)<br>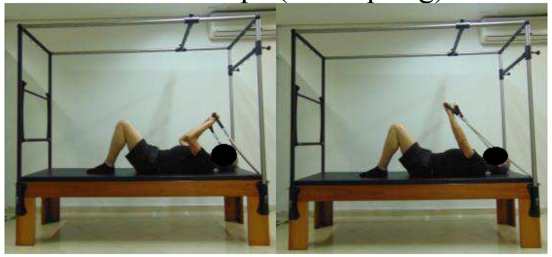<br>Objective: strengthen the triceps brachii muscle.	Arm Triceps (patient far from the spring)<br>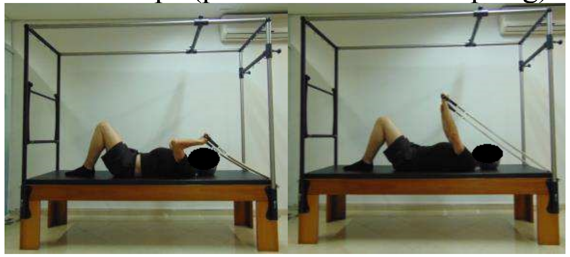<br>Objective: strengthen the triceps brachii muscle.
Triceps Sit<br>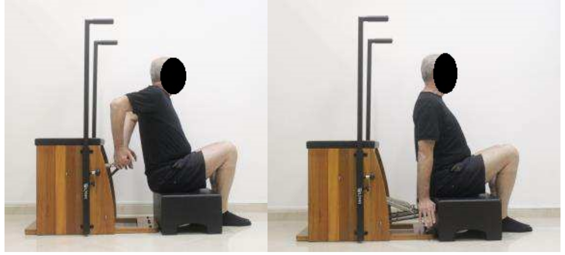<br>Objective: strengthen the triceps brachii and anconeous muscles.	Triceps Sit On Foot Bar<br>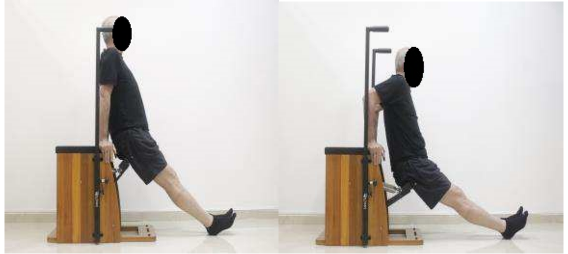<br>Objective: strengthen the triceps brachii and anconeous muscles.	Triceps Front<br>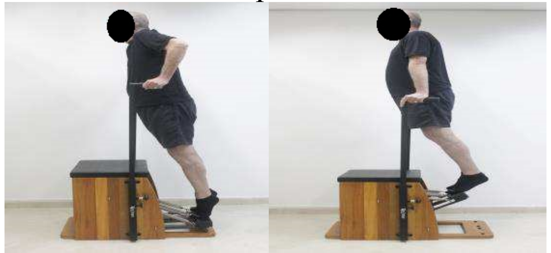<br>Objective: strengthen the triceps brachii, anconeous and pectoralis major muscles.

Abdominal Exercises		
**Basic**	**Intermediate**	**Advanced**
The Hundred Variant<br>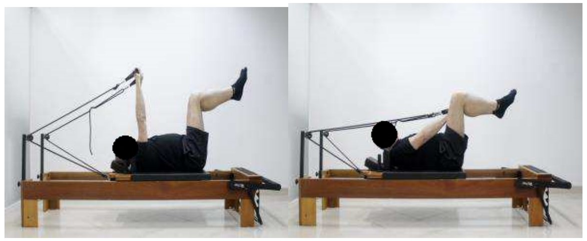<br>Objective: work coordination, stretch adductor, pectin and gracili muscles and strengthen the rectus abdominis, oblique and latissimus dorsi muscles.	The Hundred<br>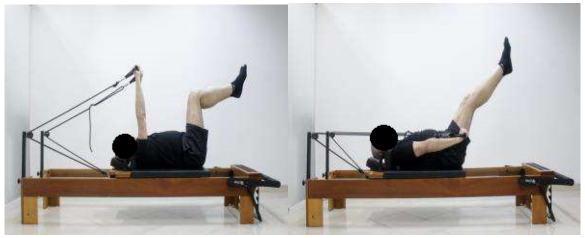<br>Objective: work coordination and strengthen the rectus abdominis, external oblique, internal oblique, latissimus dorsi, teres major and quadriceps femoris muscles.	The Hundred Variant<br>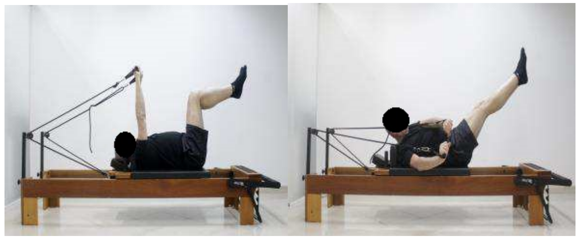<br>Objective: work coordination and strengthen the rectus abdominis, external oblique, internal oblique, latissimus dorsi, teres major and quadriceps femoris muscles.
Sit-Up<br>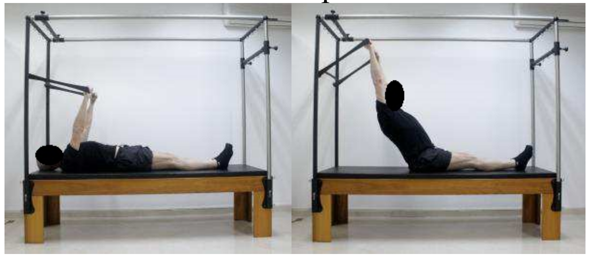<br>Objective: stretch anterior chain shoulder muscles and strengthen the rectus abdominis and external oblique muscles.	Sit-Up Variant<br>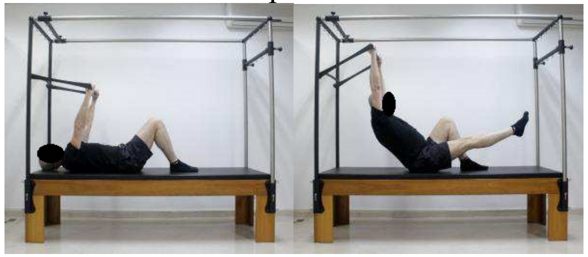<br>Objective: mobilize the spine and strengthen the rectus abdominis, external oblique and quadriceps femoris muscles.	Sit-Up Variant<br>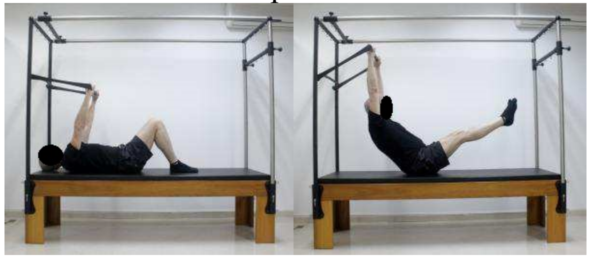<br>Objective: mobilize the spine and strengthen the rectus abdominis, external oblique, iliopsoas and quadriceps femoris muscles.
Sit-Up<br>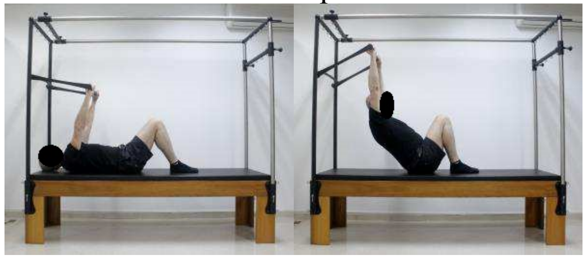<br>Objective: mobilize the spine and strengthen the rectus abdominis and external oblique muscle.	Sit-Up Variant<br>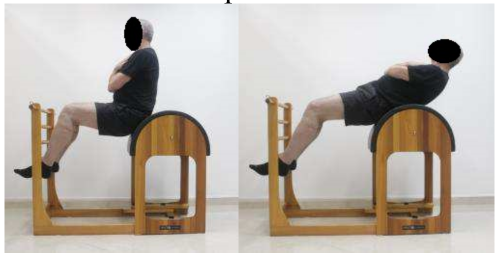<br>Objective: stretch anterior chain shoulder muscles and strengthen the rectus abdominis and external oblique muscles.	Sit-Up Variant<br>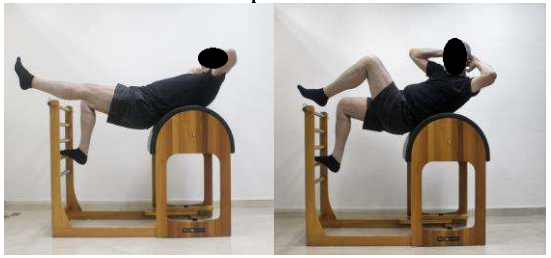<br>Objective: stretch anterior chain shoulder muscles and strengthen the rectus abdominis, external oblique, quadriceps femoris, and iliopsoas muscles.
