# Supplementary material for: Effectiveness of the Pilates method versus aerobic exercises in the treatment of older adults with chronic low back pain: a randomized controlled trial protocol
Source: BMC Musculoskelet Disord. 2019 May 24;20:250. doi: 10.1186/s12891-019-2642-9 (PMC6533704; doi:10.1186/s12891-019-2642-9)
Supplement: Supplementary file 2 — Equipment-based exercises. The appendix 2 contains photos and description of Pilates exercises that could be performed on equipment. (PDF 2285 kb) [file 12891_2019_2642_MOESM2_ESM.pdf]

APPENDIX 2- Mat based-exercises

Spine exercises		
**Basic**	**Intermediate**	**Advanced**
Bridge 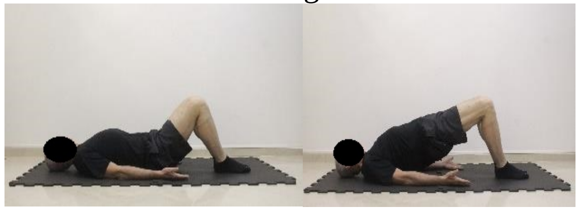 Objective: mobilize pelvis and spine and strengthen gluteus and hamstring muscles.	Bridge Variant 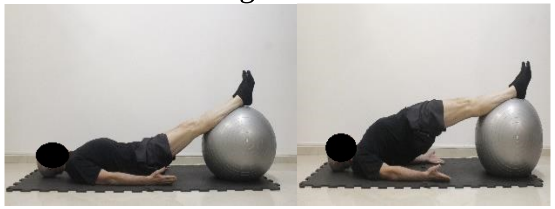 Objective: mobilize pelvis and spine, and strengthen gluteus, hamstring and gastrocnemius muscles.	Bridge Variant 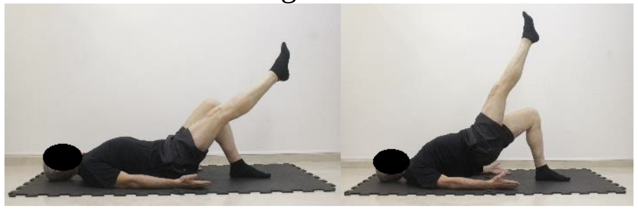 Objective: mobilize pelvis and spine and strengthen gluteus, quadriceps femoris and hamstring muscles
Rolling Back Down/Up (knees flexed and rolling forward) 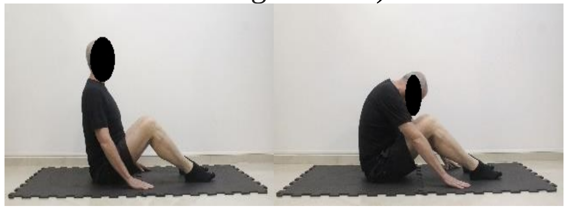 Objective: mobilize the spine and contract the abdominis muscles associated with the power house.	Rolling Back Down/Up (knees extended and rolling forward.) 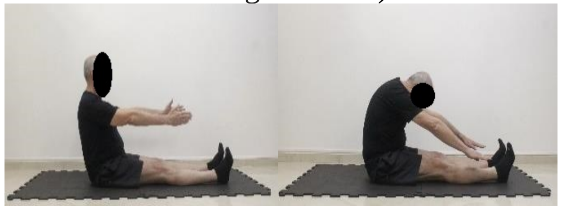 Objective: mobilize the spine and contract the abdominis muscles associated with the power house.	Roll Up (rolling back and forward) 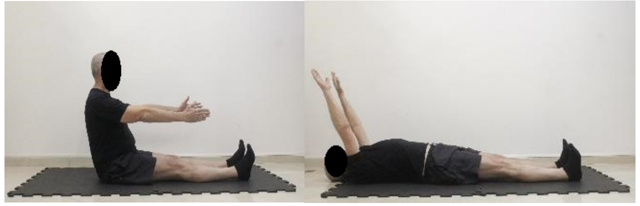 Objective: mobilize the spine and strengthen the rectus abdominis and external external oblique muscles.
Hamstring Stretch Variant 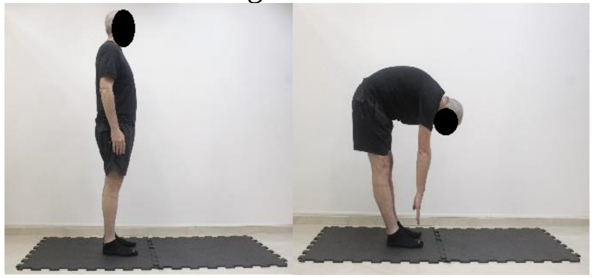 Objective: mobilize the spine and stretch the muscles of the posterior chain.	Hamstring Stretch Variant (crossed legs) 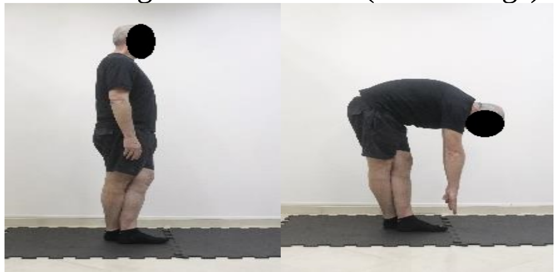 Objective: mobilize the spine and stretch the muscles of the posterior chain.	Hamstring Stretch (ball) 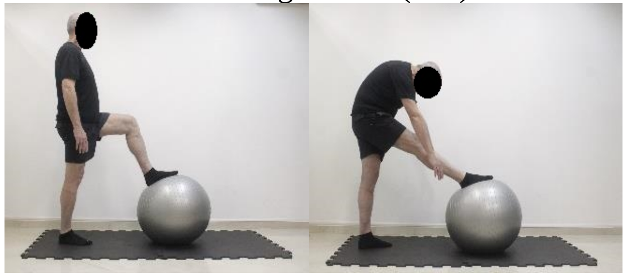 Objective: mobilize the spine and stretch the muscles of the posterior chain.

Stretches Front Variant (standing with the ball)<br>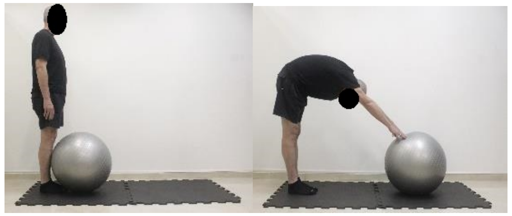<br>Objective: mobilize the spine and stretch the muscles of the posterior chain.	Hamstring Stretch Variant (sitting on the floor with one leg extended and the other one flexed)<br>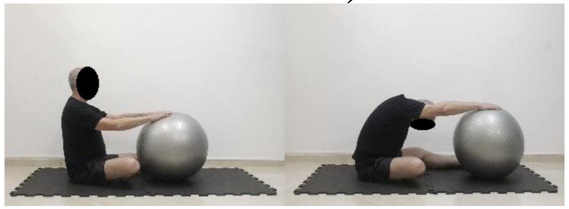<br>Objective: mobilize the spine and stretch the muscles of the posterior chain.	Spine Stretch (sitting on the floor with both legs extended)<br>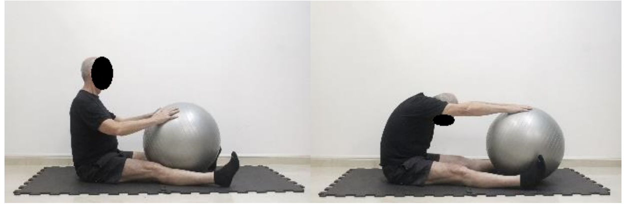<br>Objective: mobilize the spine and stretch the muscles of the posterior chain.
Swan Front<br>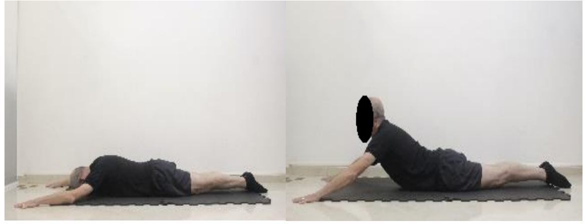<br>Objective: mobilize spine, stretching the muscles of the anterior chain, and strengthen pectoralis major, triceps brachii and anterior deltoid muscles.	Swan (taking off the arms)<br>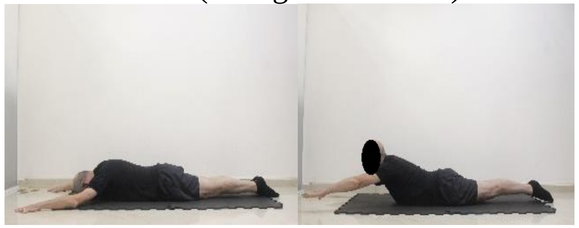<br>Objective: mobilize the spine, control the scapular elevation movement and strengthen posterior chain muscles.	Swan (taking off arms and legs alternate)<br>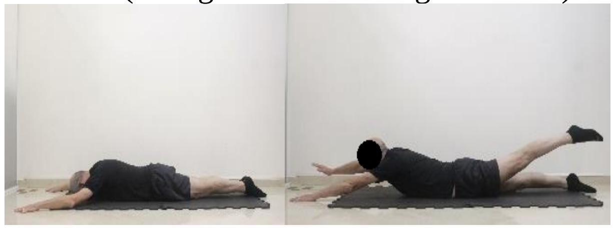<br>Objective: mobilize the spine, control the scapular elevation movement and strengthen posterior chain muscles.
Tower (in "V" position with the elastic forward and upward)<br>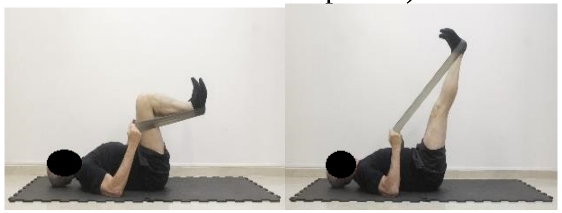<br>Objective: stretch the posterior chain muscles and strengthen the abdominis, quadriceps femoris and iliopsoas muscles.	Long Spine (in "V" position with the elastic forward and downwards)<br>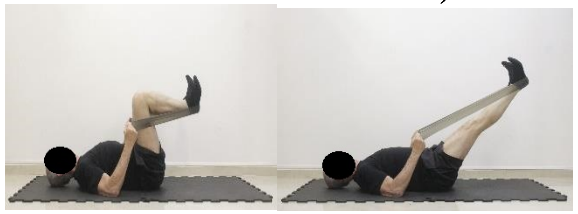<br>Objective: stretch the posterior chain muscles and strengthen the abdominis, quadriceps femoris and iliopsoas muscles.	Jackknife<br>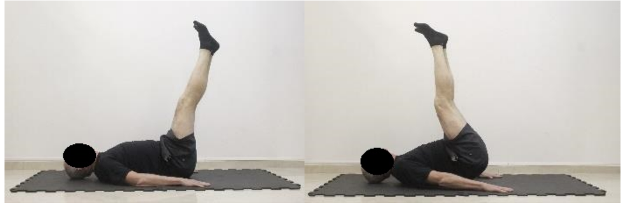<br>Objective: mobilize the spine and strengthen the abdominis, quadriceps femoris and iliopsoas muscles.

Mermaid (both hands on the ball)	Mermaid (one hand on the ball)	Side Arm Sit Variant
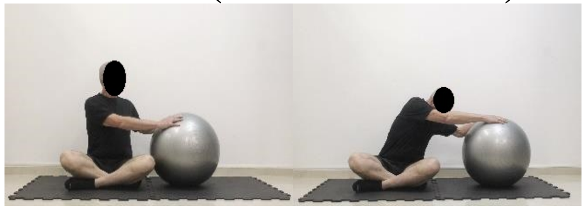	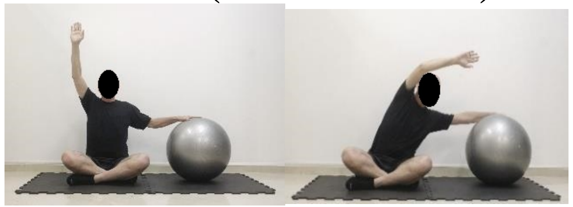	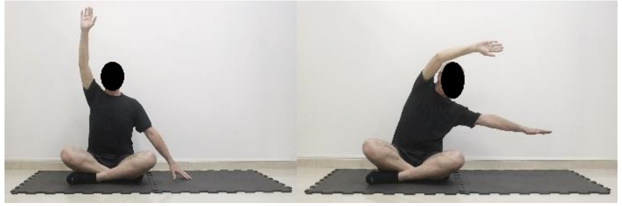
Objective: stretch lateral chain muscles and control scapular elevation movement.	Objective: stretch lateral chain muscles and control scapular elevation movement.	Objective: stretch lateral chain muscles and control scapular elevation movement.

**Lower Limbs Exercises**		
**Basic**	**Intermediate**	**Advanced**
Tower (with the elastic on one leg)<br>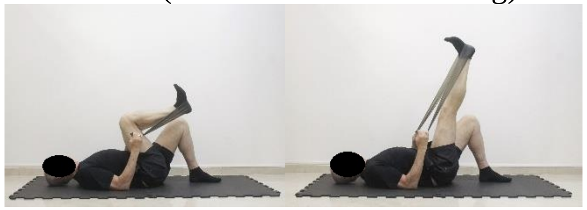<br>Objective: stretch the posterior chain muscles and strengthen the quadriceps femoris and gluteus maximus muscles.	Tower (with the elastic on both legs)<br>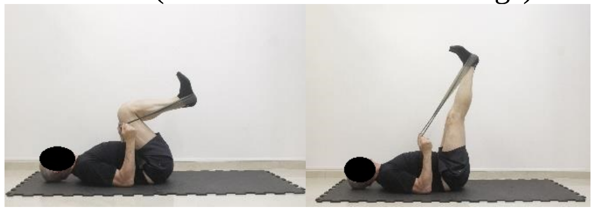<br>Objective: stretch the posterior chain muscles and strengthen the quadriceps femoris and gluteus maximus muscles.	Tower (with the elastic on one leg)<br>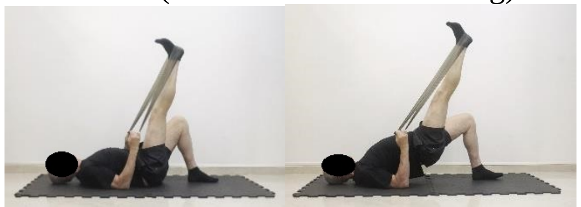<br>Objective: stretch the posterior chain muscles and strengthen the quadriceps femoris, hamstrings and gluteus maximus muscles.
Footwork (taking the foot support)<br>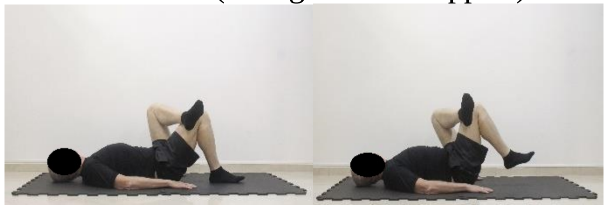<br>Objective: stretch the gluteus maximus and piriformis muscles and strengthen the quadriceps femoris muscle.	One Leg Up and Down (with the ball)<br>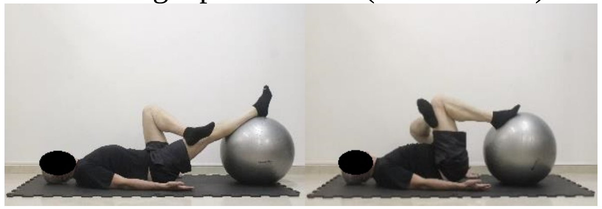<br>Objective: stretch the gluteus maximus and piriformis muscles and strengthen abdominis, iliopsoas and quadriceps femoris muscles.	Tower<br>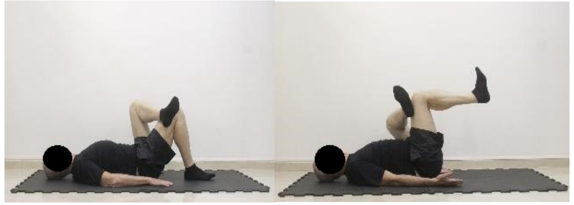<br>Objective: stretch the gluteus maximus and piriformis muscles and strengthen the quadriceps femoris muscle.
Leg Circles (small)<br>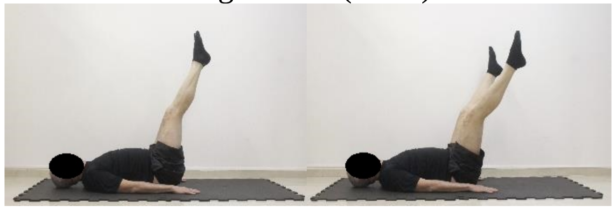<br>Objective: strengthen gluteus maximus, hamstrings, adductor magnus, adductor longus, pectineus and gracillis muscles.	Leg Circle (medium)<br>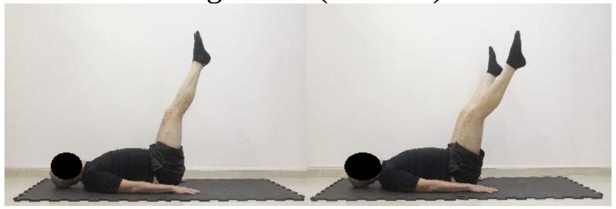<br>Objective: strengthen gluteus maximus, hamstrings, adductor magnus, adductor longus, pectineus and gracillis muscles.	Leg Circles (big)<br>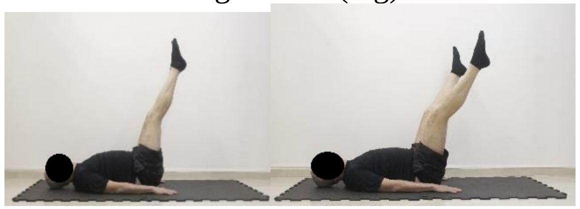<br>Objective: strengthen gluteus maximus, hamstrings, adductor magnus, adductor longus, pectineus and gracillis muscles.

Bicycle (with mild elastic)<br>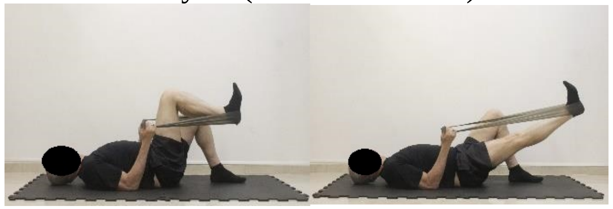<br>Objective: strengthen the gluteus maximus, quadriceps femoris, abdominis and hamstring muscles.	Bicycle (with heavy elastic)<br>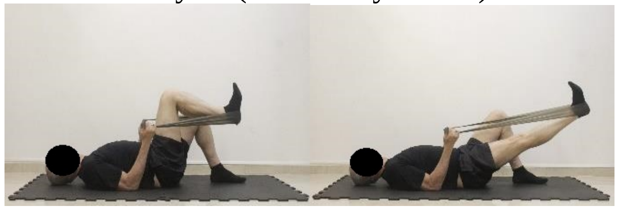<br>Objective: strengthen the gluteus maximus, quadriceps femoris, abdominis and hamstring muscles	Bicycle (both legs on the air)<br>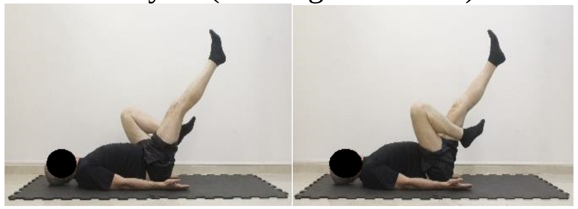<br>Objective: strengthen the gluteus maximus, hamstring, sartorius, gracillis and gastrocnemius muscles.
Footwork Hells V Position (with the elastic band)<br>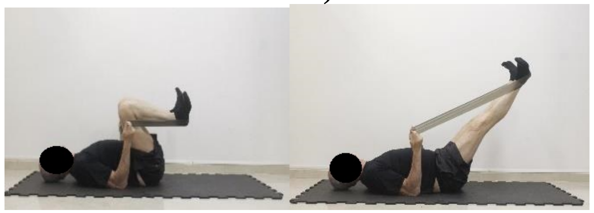<br>Objective: strengthen the quadriceps femoris, abdominis, adductor magnus, adductor longus, pectineus and gracillis muscles.	Leg Series Supine Frog (with the elastic band)<br>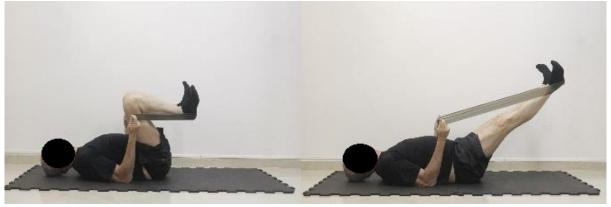<br>Objective: strengthen the quadriceps femoris, abdominis, adductor magnus, adductor longus, pectineus and gracillis muscles.	Knee Extension V Position (with the elastic band)<br>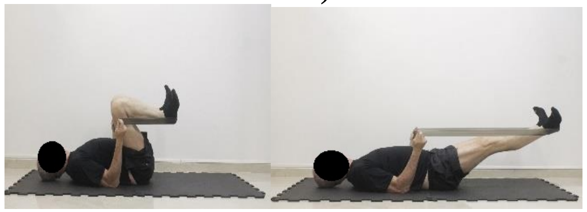<br>Objective: strengthen the quadriceps femoris, abdominis, adductor magnus, adductor longus, pectineus and gracillis muscles.
Footwork Double Legato (four supports rising one leg)<br>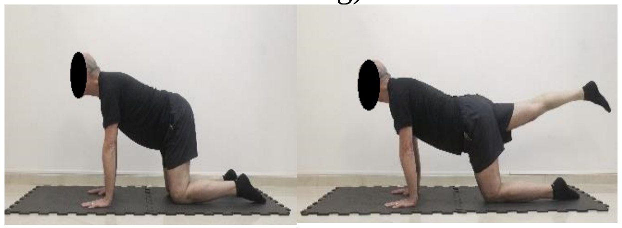<br>Objective: strengthen the quadriceps femoris, triceps surae, iliopsoas and sartorius muscles.	Footwork Double Legato (four supports with mild elastic band)<br>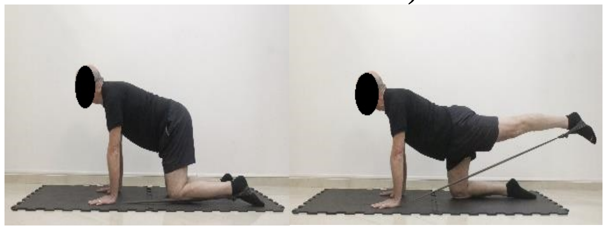<br>Objective: strengthen the quadriceps femoris, triceps surae, iliopsoas and sartorius muscles.	Footwork Double Legato (four supports with heavy elastic band)<br>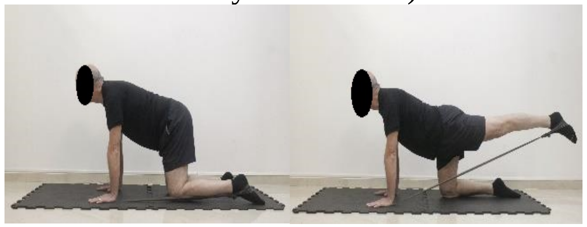<br>Objective: strengthen the quadriceps femoris, triceps surae, iliopsoas and sartorius muscles.

Side Kicks Up and Down<br>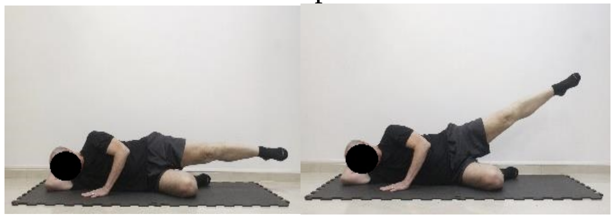<br>Objective: strengthen the gluteus medius and tensor fascia lata muscles.	Side Kicks Up and Down (mild weight)<br>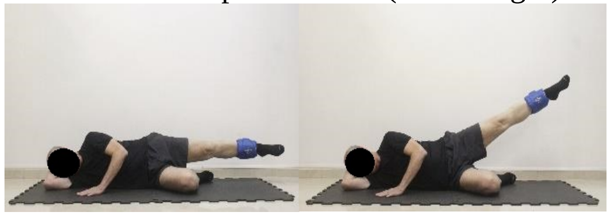<br>Objective: strengthen the gluteus medius and tensor fascia lata muscles.	Side Kicks Up and Down (heavy weight)<br>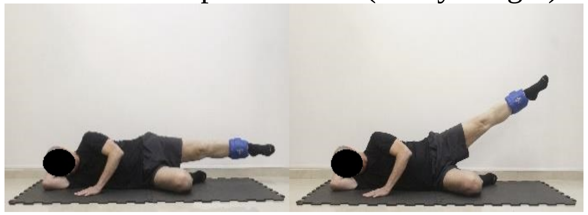<br>Objective: strengthen the gluteus medius and tensor fascia lata muscles.
Side Kicks Inner-Thigh Lifts<br>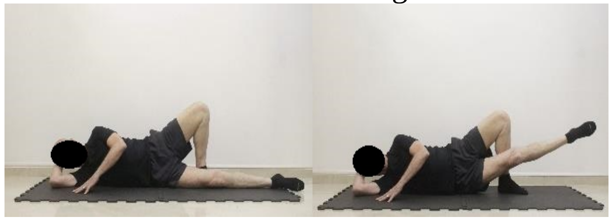<br>Objective: strengthen the gluteus maximus, hamstrings, adductor magno, adductor longus, pectineus and gracillis muscles.	Side Kicks Inner-Thigh Lifts (mild weight)<br>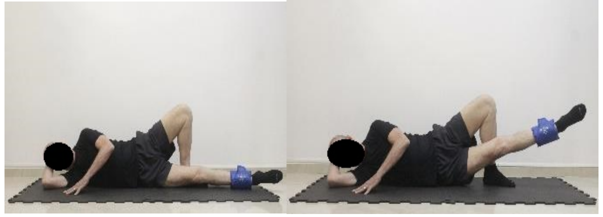<br>Objective: strengthen the gluteus maximus, hamstrings, adductor magno, adductor longus, pectineus and gracillis muscles.	Side Kicks Inner-Thigh Lifts (heavy weight)<br>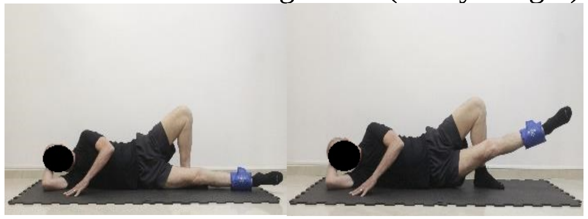<br>Objective: strengthen the gluteus maximus, hamstrings, adductor magno, adductor longus, pectineus and gracillis muscles.
One Leg Up-Down<br>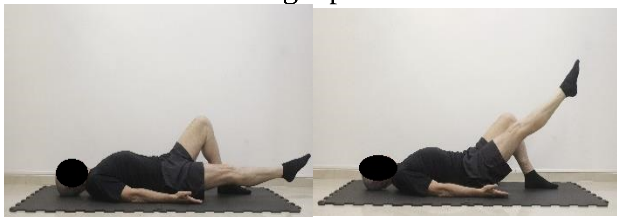<br>Objective: strengthen the iliopsoas, rectus femoris and rectus abdominis muscles.	One Leg Up-Down (mild weight)<br>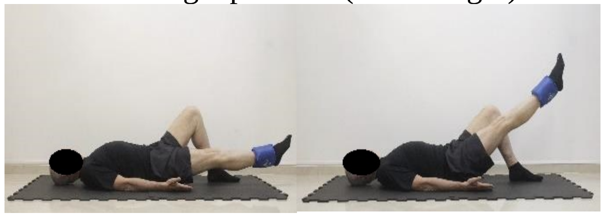<br>Objective: strengthen the iliopsoas, rectus femoris and rectus abdominis muscles.	One Leg Up-Down (heavy weight)<br>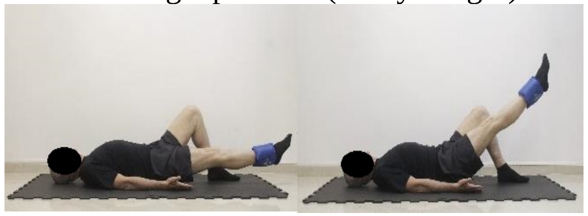<br>Objective: strengthen the iliopsoas, rectus femoris and rectus abdominis muscles.
Fire Hydrant<br>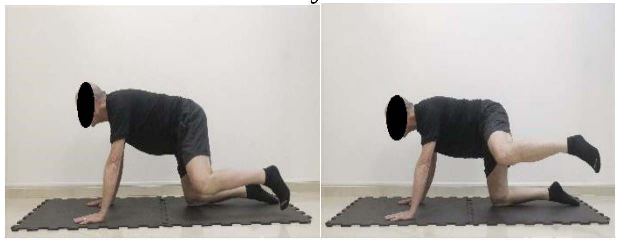<br>Objective: strengthen the quadriceps femoris and gluteus muscles.	Fire Hydrant (mild elastic)<br>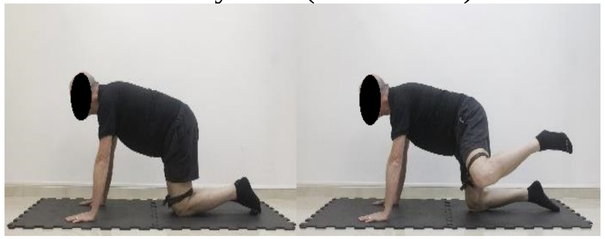<br>Objective: strengthen the quadriceps femoris and gluteus muscles.	Fire Hydrant (heavy elastic)<br>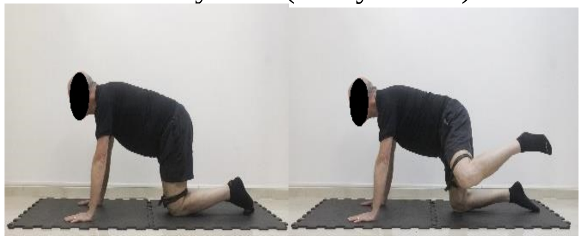<br>Objective: strengthen the quadriceps femoris and gluteus muscles.

Squatting with the ball on the wall<br>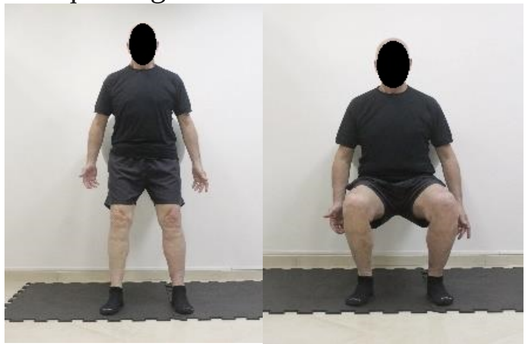<br>Objective: strengthen the quadriceps femoris and gluteus muscles.	One-legged squat with ball on wall<br>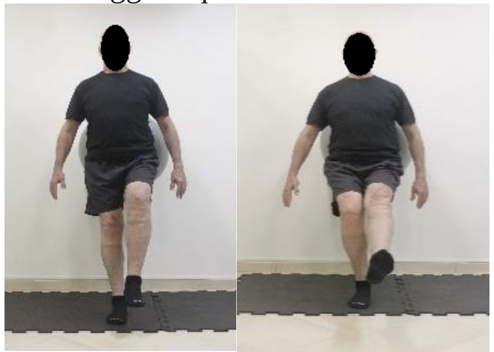<br>Objective: strengthen the quadriceps femoris and gluteus muscles.	Air squat<br>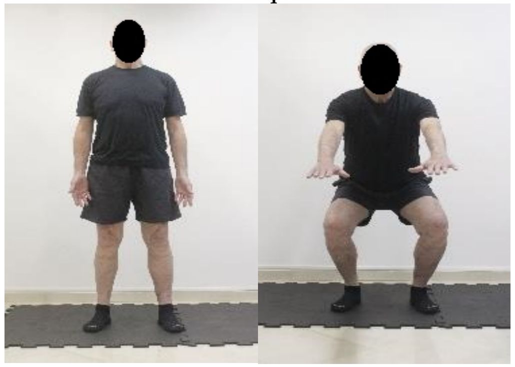<br>Objective: strengthen the quadriceps femoris and gluteus muscles.
Leg Series Diagonal<br>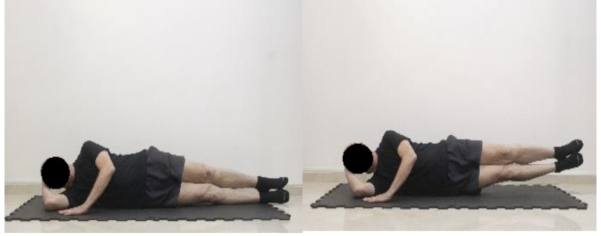<br>Objective: strengthen the gluteus medius and minimum, tensor fascia lata and sartorius muscles.	Leg Series Diagonal with circle<br>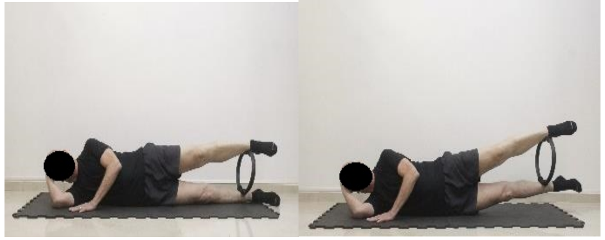<br>Objective: strengthen the gluteus medius and minimum, tensor fascia lata and sartorius muscles.	Leg Series Diagonal with the ball<br>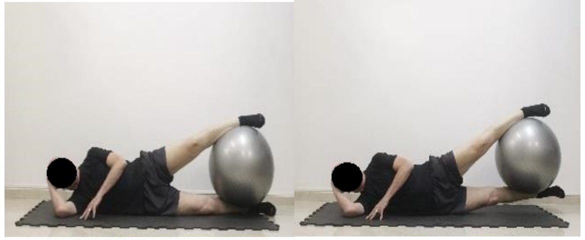<br>Objective: strengthen the gluteus medius and minimum, tensor fascia lata and sartorius muscles.
Horse<br>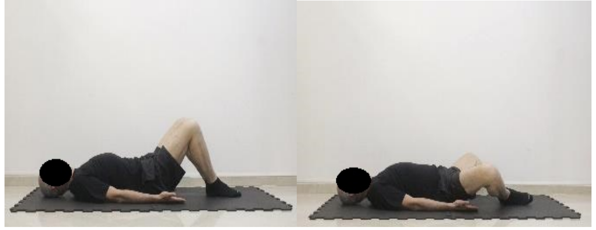<br>Objective: strengthen the abductor muscles of the hip.	Horse (mild elastic)<br>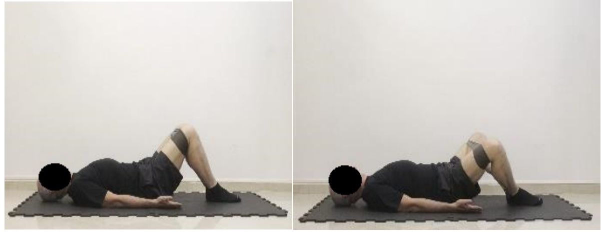<br>Objective: strengthen the abductor muscles of the hip.	Horse (heavy elastic)<br>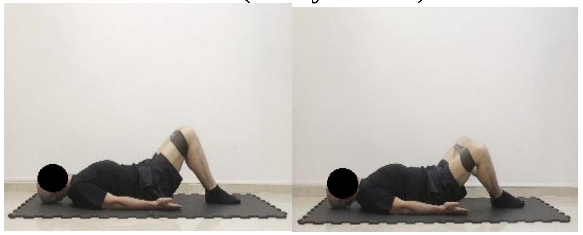<br>Objective: strengthen the abductor muscles of the hip.

<table>
<tr><th colspan="3">Upper Limbs Exercises</th></tr>
<tr><th>Basic</th><th>Intermediate</th><th>Advanced</th></tr>
<tr>
<td>Arms Biceps (with the elastic)
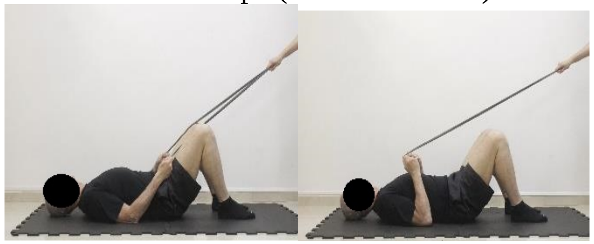
Objective: strengthen the biceps brachii, brachialis and rectus abdominis muscles.</td>
<td>Arms Biceps (flexed legs with the elastic)
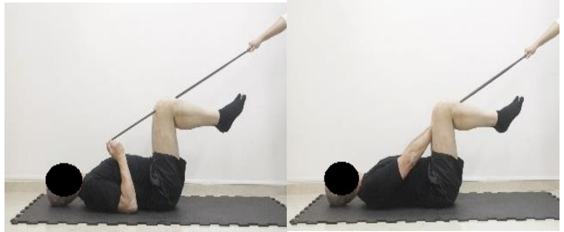
Objective: strengthen the biceps brachii, brachialis and rectus abdominis muscles.</td>
<td>Arms Biceps (standing with the elastic)
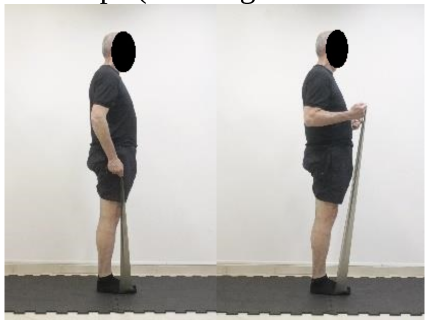
Objective: strengthen the biceps brachii, brachialis and rectus abdominis muscles.</td>
</tr>
<tr>
<td>Arms Pull Up and Down
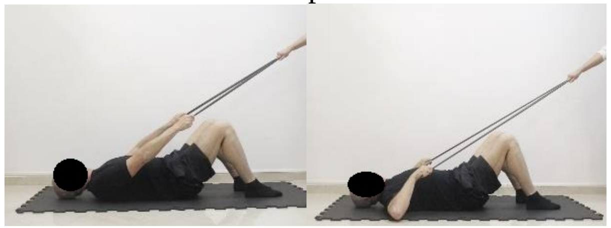
Objective: strengthen deltoid and trapezius muscles.</td>
<td>Arms Pulling (mild elastic)
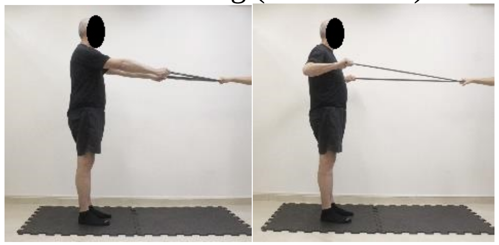
Objective: strengthen rhomboid, posterior deltoid, teres minor, infraspinatus, supraspinatus and medial and lower trapezius fibers muscles.</td>
<td>Arms Pulling Variant (heavy elastic)
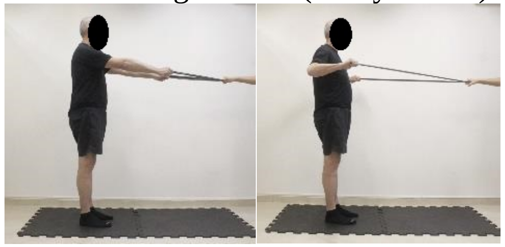
Objective: strengthen rhomboid, posterior deltoid, teres minor, infraspinatus, supraspinatus and medial and lower trapezius fibers muscles.</td>
</tr>
<tr>
<td>Arms Pull Up and Down Variant (mild elastic)
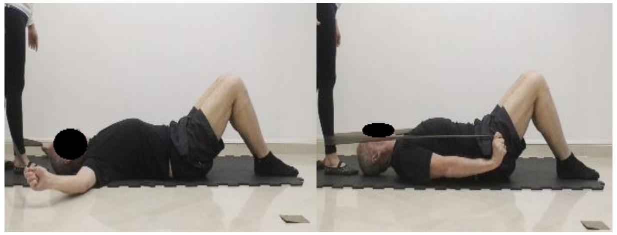
Objective: strengthen the deltoid, latissimus dorsi, teres major, pectoralis major and coracobrachialis muscles.</td>
<td>Arms Pull Up and Down Variant (flexed knees and mild elastic)
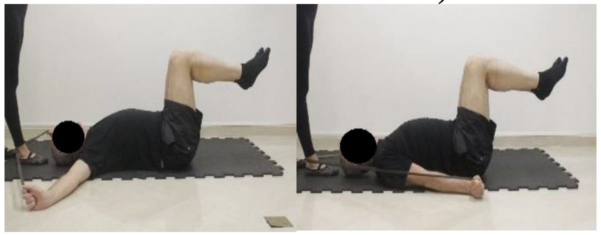
Objective: strengthen the abdominis, deltoid, latissimus dorsi, teres major, pectoralis major and coracobrachialis muscles.</td>
<td>Arms Pull Up and Down Variant (flexed knees and heavy elastic)
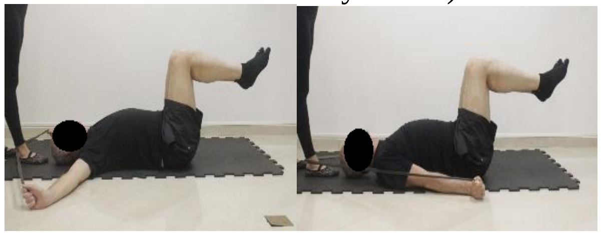
Objective: strengthen the abdominis, deltoid, latissimus dorsi, teres major, pectoralis major and coracobrachialis muscles.</td>
</tr>
</table>

Arms Triceps (mild elastic)<br>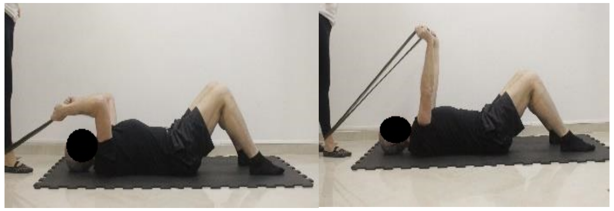<br>Objective: strengthen the triceps brachii muscle.	Arm Triceps (mild elastic without feet support)<br>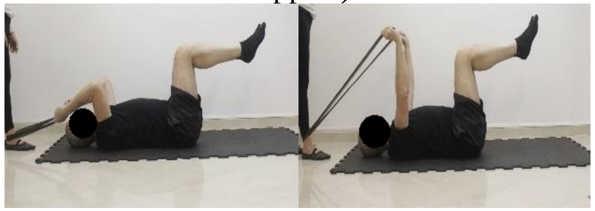<br>Objective: strengthen the triceps brachii and abdominis muscles.	Arm Triceps (heavy elastic without feet support)<br>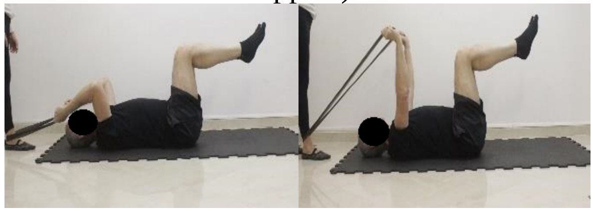<br>Objective: strengthen the triceps brachii and abdominis muscles.

**Abdominal Exercises**		
**Basic**	**Intermediate**	**Advanced**
Teaser<br>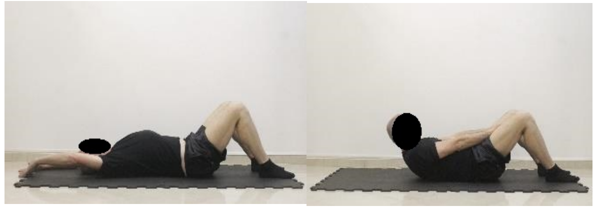<br>Objective: stretch the adductor magnus, adductor longus, adductor minimum, pectineus and gracillis muscles and strengthen the rectus abdominis and external oblique muscles.	The Hundred Variant (with feet on the air)<br>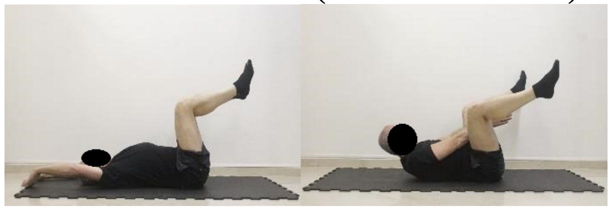<br>Objective: work coordination, stretch adductor, pectineus and gracillis muscles and strengthen the rectus abdominis, external oblique, latissimus dorsi and teres major muscles.	Exercise On the Ball<br>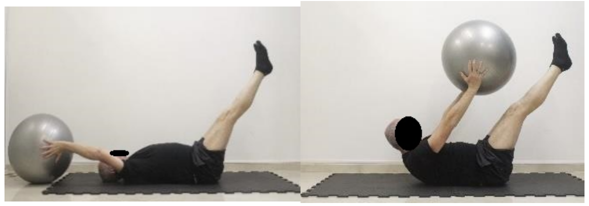<br>Objective: work coordination, stretch adductor, pectineus and gracillis muscles and strengthen the rectus abdominis, external oblique, latissimus dorsi and teres major muscles.
The Hundred (pumping arms and stretching legs)<br>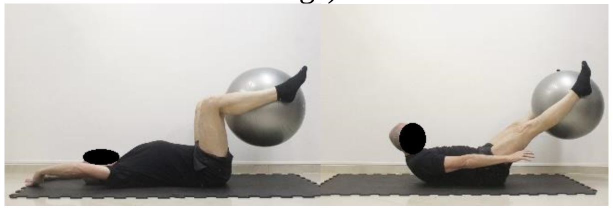<br>Objective: work coordination and strengthen the rectus abdominis and external oblique muscles.	The Hundred (raising the ball)<br>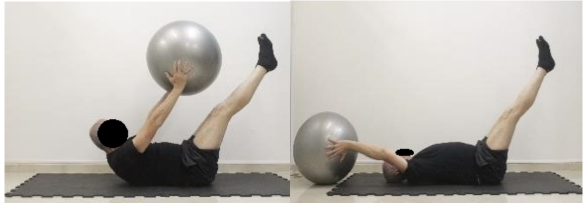<br>Objective: work coordination and strengthen the rectus abdominis and external oblique muscles.	The Hundred Variant (side)<br>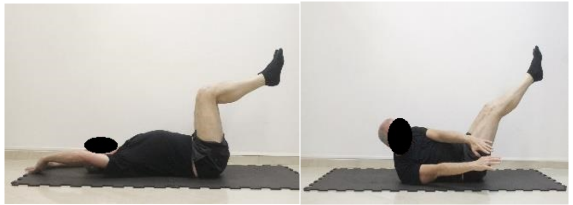<br>Objective: work coordination and strengthen the rectus abdominis and external oblique muscles.

Bridge with knees and feet supported	Bridge with knees supported	Bridge
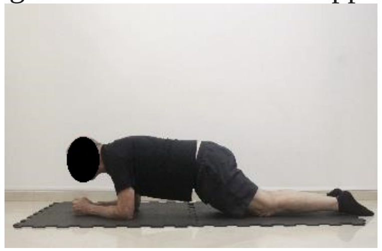	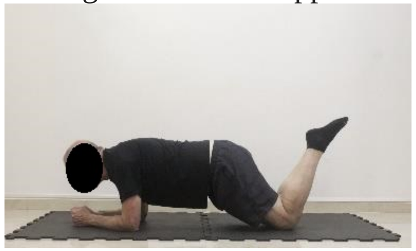	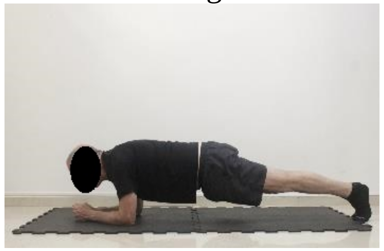
Objective: strengthen the abdominis rectus, internal oblique, external oblique, iliopsoas, pectoral and triceps brachii muscles.	Objective: strengthen the abdominis rectus, internal oblique, external oblique, iliopsoas, pectoral and triceps brachii muscles.	Objective: strengthen the abdominis rectus, internal oblique, external oblique, iliopsoas, pectoral and triceps brachii muscles.
